# Patterns of spontaneous and induced genomic alterations in *Yarrowia lipolytica*

**DOI:** 10.1128/aem.01678-24

**Published:** 2024-12-23

**Authors:** Yuan-Ru Xiong, Yuan-Chun Fang, Min He, Ke-Jing Li, Lei Qi, Yang Sui, Ke Zhang, Xue-Chang Wu, Liang Meng, Ou Li, Dao-Qiong Zheng

**Affiliations:** 1 College of Life Sciences and Medicine, Zhejiang Sci-Tech University 98445 , Hangzhou, China; 2 Ocean College, Zhejiang University 601090 , Zhoushan, China; 3 College of Life Science, Zhejiang University 12377 , Hangzhou, China; 4 BGI Research 672740 , Sanya, China; Chalmers tekniska hogskola AB, Gothenburg, Sweden

**Keywords:** genomic alterations, Zeocin, mutation spectrum, drug resistance, *Yarrowia lipolytica*

## Abstract

This study explored the genomic alterations in *Yarrowia lipolytica*, a key yeast in industrial biotechnology, under both spontaneous and mutagen-induced conditions. Our findings reveal that spontaneous mutations occur at a rate of approximately 4 × 10^−10^ events per base pair per cell division, primarily manifesting as single-nucleotide variations (SNVs) and small insertions and deletions (InDels). Notably, C-to-T/G-to-A transitions and C-to-A/G-to-T transversions dominate the spontaneous SNVs, while 1 bp deletions, likely resulting from template slippage, are the most frequent InDels. Furthermore, chromosomal aneuploidy and rearrangements occur, albeit at a lower frequency. Exposure to ultraviolet (UV) light, methylmethane sulfonate (MMS), and Zeocin significantly enhances the rates of SNVs and alters their mutational spectra in distinct patterns. Notably, Zeocin-induced SNVs are predominantly T-to-A and T-to-G substitutions, often occurring within the 5′-TGT^*^−3′ motif (^*^ denotes the mutated base). Additionally, Zeocin exhibits a higher potency in stimulating InDels compared to UV and MMS. Translesion DNA synthesis is implicated as the primary mechanism behind most Zeocin-induced SNVs and some InDels, whereas non-homologous end joining serves as the main pathway for Zeocin-mediated InDels. Intriguingly, the study identifies the gene *YALI1_E21053g*, encoding a protein kinase, as negatively associated with Zeocin resistance. Overall, our results not only deepened our knowledge about the genome evolution in *Y. lipolytica* but also provided reference to develop innovative strategies to harness its genetic potential.

**IMPORTANCE:**

*Yarrowia lipolytica* exhibits high environmental stress tolerance and lipid metabolism capabilities, making it a microorganism with significant industrial application potential. In this study, we investigated the genomic variation and evolutionary patterns of this yeast under both spontaneous and induced mutation conditions. Our results reveal distinctive mutation spectra induced by different mutagenic conditions and elucidate the underlying genetic mechanisms. We further highlight the roles of non-homologous end joining and translesion synthesis pathways in Zeocin-induced mutations, demonstrating that such treatments can rapidly confer drug resistance to the cells. Overall, our research enhances the understanding of how yeast genomes evolve under various conditions and provides guidance for developing more effective mutagenesis and breeding techniques.

## INTRODUCTION

Yeasts are single-celled eukaryotes that are generally straightforward to culture in laboratory settings. Among them, certain species, such as *Saccharomyces cerevisiae*, emerged as prominent model organisms in life science research. In addition to their research applications, yeast strains are also crucial in industrial biotechnology due to their unique advantages (1–5). The oleaginous yeast *Yarrowia lipolytica*, for instance, has adapted to thrive in a variety of environments, including soil, sewage, and oil-contaminated sites (6). This yeast is particularly notable for its tolerance to various organic compounds, pH levels, and high salt concentrations. Additionally, it possesses the ability to assimilate a diverse array of both hydrophobic and hydrophilic carbon sources (7, 8). Different strains of *Y. lipolytica* exhibit distinct metabolic and phenotypic traits based on their resources and environmental backgrounds (9–11). Comparative genomic studies have revealed significant sequence divergence among *Y. lipolytica* strains (12), though the underlying genetic mechanisms responsible for these genomic alterations remain to be fully elucidated.

Spontaneous mutations serve as a fundamental source of genetic variation, playing a crucial role in the adaptation of organisms to new selective pressures. Research utilizing *S. cerevisiae* has documented a variety of spontaneous genomic alterations, including single-nucleotide variations (SNVs), small insertions and deletions (InDels), loss of heterozygosity, chromosomal rearrangements, and whole-chromosome aneuploidy. Notably, both the mutation rate and the spectrum of mutations—referring to the types of base substitutions—can be significantly influenced by external stressors such as ultraviolet (UV) light (13–15), methylmethane sulfonate (MMS) (16, 17), phleomycin (Zeocin) (18, 19), hydrogen peroxide (20, 21), and furan derivatives (22, 23). Furthermore, these studies have shown that certain genetic events are not randomly distributed across chromosomes. In contrast to *S. cerevisiae*, the non-conventional yeast *Y. lipolytica* possesses a larger genome with higher GC content, dispersed 5S ribosomal RNA genes (24–26), signal recognition particle-type 7SL RNA sequences (27, 28), a higher proportion of non-coding regions (1, 29), and the presence of various transposable elements (24, 30). Understanding how genomic alterations occur under both spontaneous and induced conditions will enhance our knowledge of biological evolution and provide valuable insights for developing breeding strategies for industrial microorganisms.

In this study, we investigated spontaneous and mutagen-induced genomic alterations in *Y. lipolytica* using mutation accumulation experiments (MAEs) combined with next-generation whole-genome sequencing. We revealed that the exposure to UV, MMS, and Zeocin, three widely used DNA damaging agents in biological studies, resulted in distinctly different mutation spectra in the *Y. lipolytica* genome. Additionally, our findings indicate that non-homologous end joining (NHEJ) and DNA translesion synthesis (TLS) are the primary pathways responsible for the unique mutation signatures associated with Zeocin exposure. We further explored the variations in genomic alteration patterns between *Y. lipolytica* and *S. cerevisiae*, discussing the potential underlying genetic mechanisms involved. Notably, we demonstrated that *Y. lipolytica* strains are likely to develop resistance to Zeocin through the deactivation of a putative kinase-encoding gene. Our results offer new insights into the mechanisms of genomic evolution in *Y. lipolytica* under both spontaneous and mutagens-induced conditions.

## RESULTS

### Whole-genome sequencing revealed genomic alterations of *Y. lipolytica*

**Fig 1:**
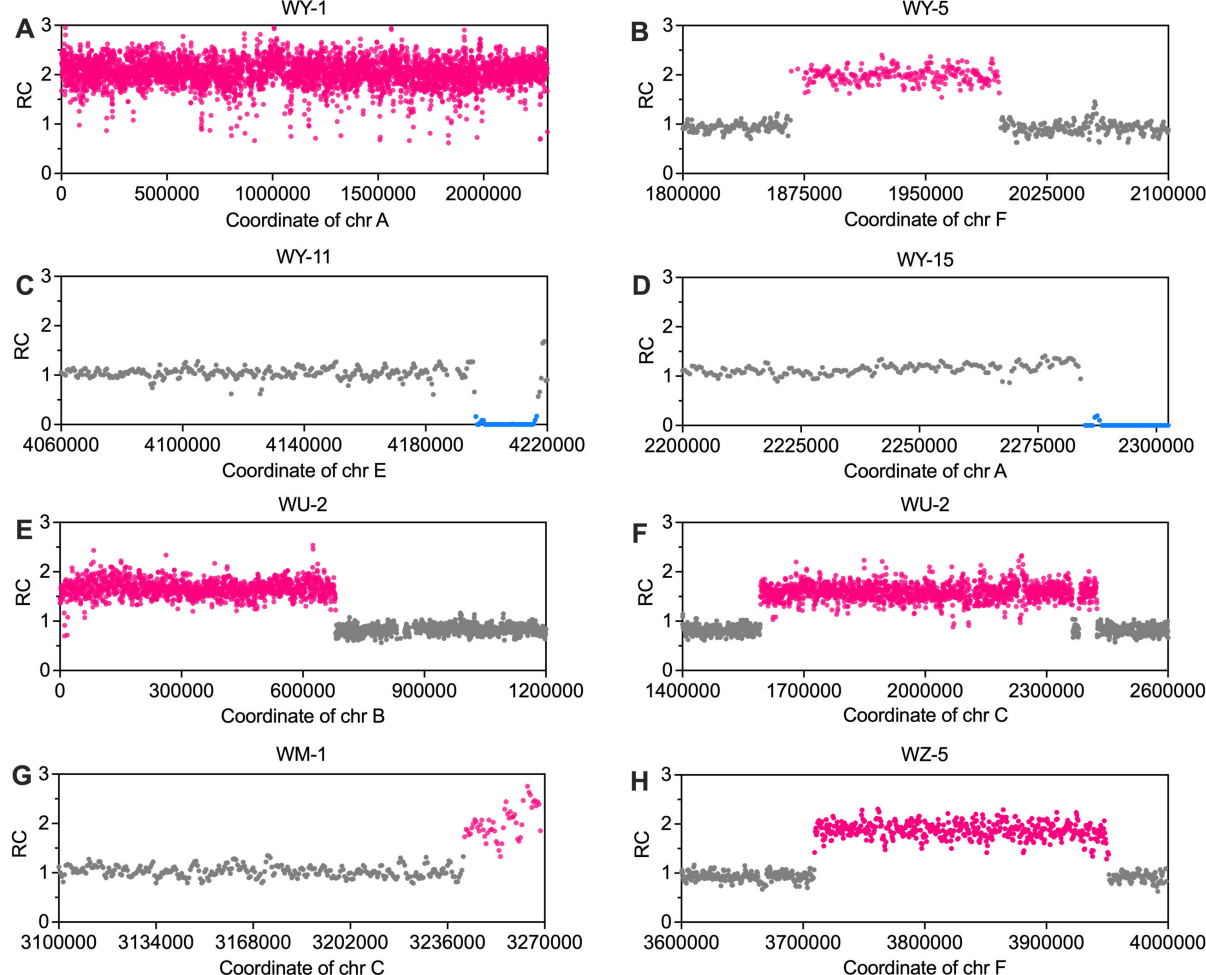
Chromosomal rearrangements and aneuploidy event in subcultured isolates of *Y. lipolytica*. Red and blue lines indicate duplicated and deleted regions, respectively. The relative coverage (RC) values of 0, 1, and 2 correspond to 0, 1, and 2 copies of DNA. (**A**) Whole-chromosome duplication in isolate WY-1. (**B**) An interstitial duplication event in isolate WY-5. Terminal deletions in isolates WY-11 (**C**) and WY-15 (**D**). A terminal duplication (**E**) and two interstitial duplications (**F**) in the UV-treated isolate WU-2. A terminal duplication event in the MMS-treated isolate WM-1 (**G**) and an interstitial duplication in the Zeocin-treated isolate WZ-5 (**H**).

To explore the patterns of spontaneous and mutagen-induced genomic alterations at the whole-genome level, we subcultured the isolates *of Y. lipolytica* W29 under both spontaneous and mutagen-treated conditions. W29 is a commonly used wild-type reference strain of *Y. lipolytica*, with its genome sequence published in 2016 (12). The isolates of W29 were grown on yeast extract peptone dextrose (YPD) medium at 30°C for spontaneous conditions and treated with UV light, MMS, or Zeocin for mutagen-induced conditions. MAE were typically conducted on solid media to minimize natural selection by randomly picking colonies from plates (31–33). The subcultured isolates were subsequently sequenced to identify genetic events at the whole-genome level. Spontaneous DNA point mutations in yeast occur at a low rate, approximately 10^−10^ per base per cell division (31, 32). As a result, repeated subculturing (typically over 100 generations) is necessary to accumulate a sufficient number of mutations (around 10) for analysis. Among the 21 isolates (designated WY1–21) grown under spontaneous conditions for either 103 generations (11 isolates) or 120 generations (10 isolates), we identified 247 SNVs and 192 small InDels (Data set S1). Of the 247 SNVs, 91 occurred in protein-coding regions: 54 were missense variants; 32 were synonymous variants; and 5 resulted in stop-codon loss (Data set S1). A minority of the InDels (9%) were intragenic, including 11 frameshift variants and 7 conservative or disruptive in-frame insertions or deletions (Data set S1). A significant portion of the identified InDels, specifically 82% (157 out of 192), were found within one-base or multiple-base tandem repeats, indicating that these mutations may be linked to template slippage during DNA replication (Data set S2). Given that a colony on a YPD plate originates from approximately 25 cell divisions, we calculated the total number of divisions during the subculture process as follows: (103 × 11 + 120 × 10) × 25 = 58,325. The *Y. lipolytica* genome size is 20,500,000 bp. Consequently, the spontaneous mutation rates for SNVs and InDels in *Y. lipolytica* were calculated to be 2.07 × 10⁻^10^ (247/58,325/20,500,000; 95% confidence interval [CI]: 1.71–2.43 × 10⁻^10^) and 1.61 × 10⁻^10^ (95% CI: 1.11–2.01 × 10⁻^10^) events per base per cell division, respectively. In addition, we identified one whole-chromosome aneuploidy (duplication of chromosome A) (Fig. 1A) and three chromosomal rearrangements (one large duplication and two large deletions) (Fig. 1B through D) among the 21 isolates. These chromosomal alterations occurred at rates of 1.71 × 10⁻^5^ events per cell division for aneuploidy and 5.14 × 10⁻^5^ (95% CI: 0–11.80 × 10⁻^5^) events per cell division for chromosomal rearrangements.

Although UV light, MMS, and Zeocin are well known for their mutagenic capabilities in enhancing desirable traits in microorganisms (34–36), their genetic effects on *Y. lipolytica* were previously uncharacterized. To investigate these effects, we independently treated nine W29 isolates with 80 J/m² UV light, eight with 0.03% MMS, or eight with 60 µg/mL Zeocin for several cycles, and sequenced them as described in the Materials and Methods section. The rates of genetic events under each treatment condition are summarized in Table 1. Our results revealed that UV and MMS treatments significantly increased the frequency of SNVs by at least an order of magnitude compared to spontaneous conditions, although their impact on InDels was relatively moderate (Table 1; Data sets S3 and S4). In contrast, Zeocin treatment led to a markedly higher rate of InDels (4.01 × 10⁻^9^ events per base per cell division, 95% CI: 2.65–5.06 × 10⁻^9^), which was 25 times greater than the spontaneous rate (Table 1; Data set S5). Interestingly, we observed that the ratio of SNVs in coding versus non-coding regions varies, depending on the treatment conditions (Fig. S2). Specifically, UV and MMS treatments resulted in higher ratios of SNVs in coding regions compared to non-coding regions, with 40% and 45% of SNVs occurring in coding regions, respectively, under these conditions (Fig. S2). Additionally, we observed several large deletions and duplications in isolates treated with these mutagens (Table 1; Fig. 1E through G). Among the mutagens tested, UV light appeared to be the most effective at inducing chromosomal rearrangements in *Y. lipolytica*, with a rate of 5.77 × 10⁻^4^ (95% CI: 0–44.1 × 10⁻^5^) events per cell division. However, it is important to note that this conclusion is based on a limited number of observed events. In summary, our data provide insight into the extent of genomic alterations in *Y. lipolytica* under both spontaneous and mutagen-treated conditions.

**TABLE 1:** Rates of genetic events in *Y. lipolytica* under spontaneous and mutagen-induced conditions

Conditions	SNVs^*a*^	InDels	Rearrangements
Spontaneous	2.07 (1.71–2.43) E-10	1.61 (1.11–2.01) E-10	5.14 (0–11.80) E-5
MMS	6.91 (4.38–8.61) E-9	2.32 (0.35–4.24) E-10	3.39 (0–12.94) E-4
UV	11.04 (0.91–1.25) E-8	6.75 (3.64–8.69) E-10	5.77 (0–44.1) E-4
Zeocin	5.32 (3.73–6.49) E-9	4.01 (2.65–5.06) E-9	1.64 (0–7.65) E-4

^*a*^ The values in parentheses indicate the 95% CI. The unit of the rates was per base per cell division.

### Distinct spectrum of spontaneous and induced SNVs

**Fig 2:**
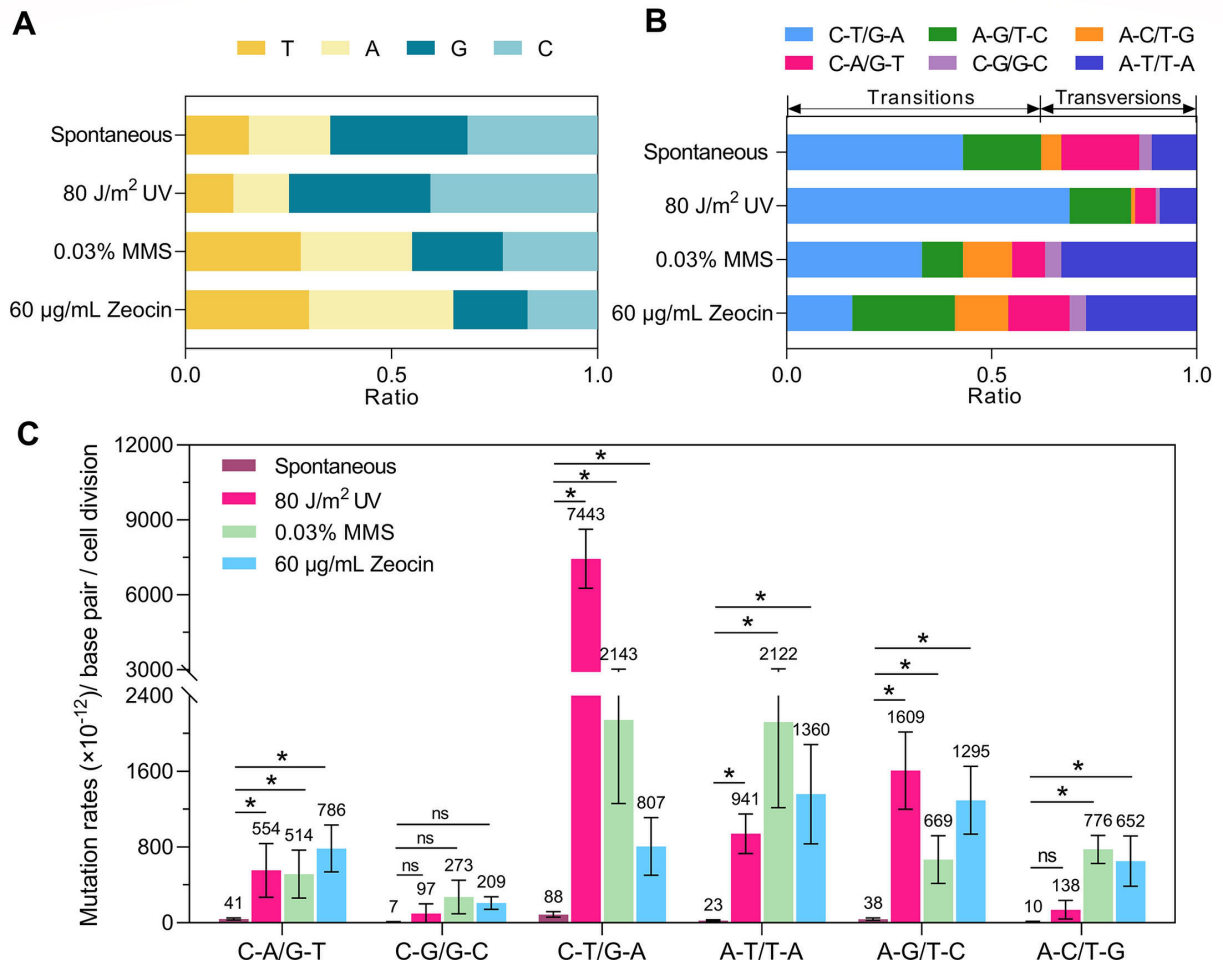
Distinct patterns of spontaneous and mutagen-induced SNVs. (**A**) Ratios of base substitutions at A, T, C, and G sites in *Y. lipolytica* isolates subcultured under spontaneous and mutagen-treated conditions. (**B**) Ratios of transition and transversion mutations. (**C**) Mutation rates of different classes of base substitutions. The data are shown as the means ± SDs. Numbers above the bars are absolute mutation rates. *P* values were determined using the Wilcoxon rank-sum test and are indicated with asterisks. *P < 0.05. ns, not significant.

More than half (65%) of the spontaneous SNVs observed in the *Y. lipolytica* genome occurred at cytosine (C) or guanine (G) bases (Fig. 2A), which is significantly higher than the expected GC ratio of 49.4% (*P* < 0.05, Fisher’s exact test). Among these, C-to-T/G-to-A transitions and C-to-A/G-to-T transversions constituted up to 43% and 19% of all base substitutions, respectively (Fig. 2B). Similar to observations in *S. cerevisiae*, the ratio of transitions to transversions is higher for spontaneous SNVs in the *Y. lipolytica* genome. UV treatment increased the frequency of each type of SNVs by 14- to 84-fold, with the most pronounced effect observed for the C-to-T/G-to-A base substitution (Fig. 2C). Consequently, the relative ratio of C–T:G–A in UV-treated cells was significantly higher compared to spontaneous SNVs (Fig. 2B; *P* < 0.05, Fisher’s exact test). In MMS- and Zeocin-treated cells, base substitutions predominantly involved adenine (A) or thymine (T) (Fig. 2A). Specifically, the rates of A-to-C/T-to-G and A-to-T/T-to-A substitutions were increased by 77- and 91-fold, respectively, in MMS-treated cells compared to spontaneous conditions (Fig. 2C). Zeocin treatment resulted in significantly higher ratios of A-to-G/T-to-C and A-to-T/T-to-A transversions compared to spontaneous SNVs (Fig. 2B; *P* < 0.05, Fisher’s exact test). Overall, our results demonstrate that UV, MMS, and Zeocin treatments significantly alter the spectrum of base substitutions at the whole-genome level.

**Fig 3:**
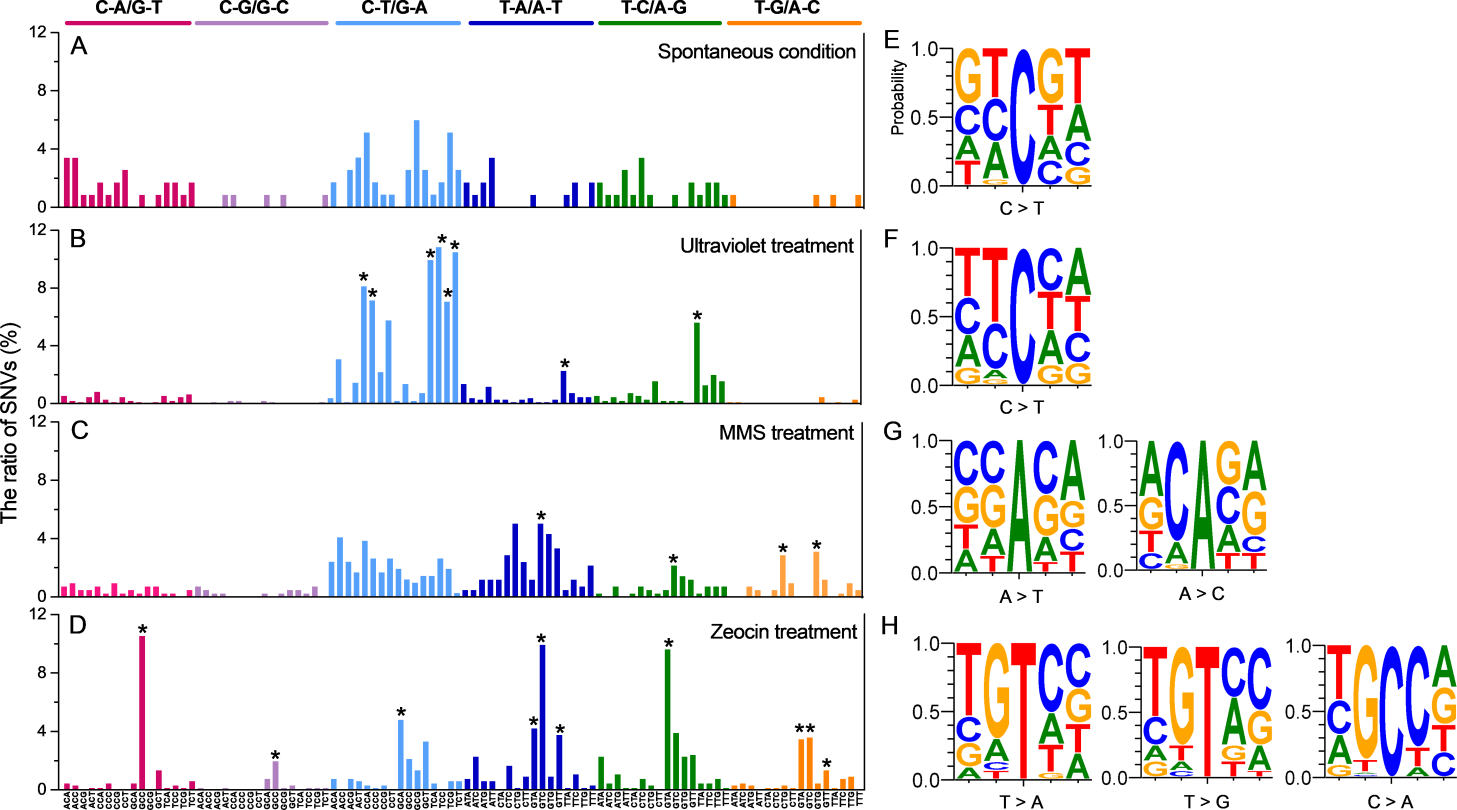
DNA sequence context of signature mutations. Adjacent sequences of mutated bases under (**A**) spontaneous, (**B**) UV treatment, (**C**) MMS treatment, and (**D**) Zeocin treatment conditions. Single-nucleotide variants (SNVs) are positioned in the middle of the three-nucleotide sequence. *P* values were determined using the chi-squared test and are indicated with asterisks. *P < 0.01. Adjacent four-base sequences for prominent base substitutions are shown for (**E**) spontaneous, (**F**) UV treatment, (**G**) MMS treatment, and (**H**) Zeocin treatment conditions, generated using WebLogo 3.

To investigate whether SNVs are influenced by surrounding sequences, we extracted trinucleotide sequences with the SNV site positioned in the center from the *Y. lipolytica* genome. If SNVs are not affected by adjacent bases, the observed frequency of trinucleotide sequences containing SNVs should be close to the expected frequency based on the genome sequence context. For spontaneous SNVs, none of the observed frequencies of trinucleotide sequences containing SNVs were significantly enriched relative to the expected ratios (*P* > 0.05, adjusted for multiple comparisons, chi-squared test) (Fig. 3A). In contrast, UV-induced SNVs showed significant context dependency. Since UV light directly causes more lesions and mutations at pyrimidine sites, G-to-A substitutions actually reflect C-to-T substitutions on the complementary strand. These substitutions were found to be more likely to occur within the sequences 5′-CCA-3′, 5′-CCC-3′, 5′-TCA-3′, 5′-TCC-3′, 5′-TCG-3′, and 5′-TCT-3′ in cells exposed to UV treatment (Fig. 3B). Additionally, the sequence 5′-TTA-3′ was enriched for UV-induced T-to-C and T-to-A substitutions (Fig. 3B). MMS-induced T-to-C and T-to-A substitutions were preferentially observed within the sequence 5′-GTC-3′, while T-to-G substitutions were more frequent in 5′-CTG-3′ and 5′-GTG-3′ (Fig. 3C). In Zeocin-treated isolates, the sequence 5′-GTA-3′ was preferred for T-to-A, T-to-C, and T-to-G substitutions, with 5′-GTC-3′ and 5′-GTT-3′ being particularly favored for T-to-A and T-to-G substitutions (*P* < 0.01, adjusted for multiple comparisons, chi-squared test). For C base SNVs in Zeocin-treated cells, C–A and C–T were the most frequent, showing biases for the sequences 5′-GCC-3′ and 5′-GCA-3′, respectively (Fig. 3D; Table S2).

We also analyzed the frequency of adjacent 4-base sequences for prominent base substitutions in spontaneous and mutagen-induced SNVs using WebLogo 3. For spontaneous conditions, no specific base enrichment was observed adjacent to the primary SNVs, which were predominantly C-to-T substitutions (Fig. 3E; Table S2). In contrast, although the major SNVs induced by UV exposure were also C-to-T substitutions, approximately 90% of the bases immediately upstream (5′) of the SNV site were pyrimidines, with C accounting for 34% and T for 55%. This distribution is significantly higher than expected by chance (Fig. 3F; Table S2). For A-to-C substitutions induced by MMS, the most frequent base immediately upstream (5′) of the SNV site was C, accounting for up to 78%. However, no bias was observed for MMS-induced A-to-T mutations (Fig. 3G). In Zeocin-treated cells, C-to-A, T-to-A, and T-to-G substitutions were most prevalent (Fig. 3D). The WebLogo results indicated that T-to-A and T-to-G substitutions tended to occur at the motif 5′-TGT*−3′ (* indicating the SNV) (Fig. 3H; Table S2). Interestingly, C-to-A substitutions were more likely to occur at the motif 5′-TGC*C-3′. These patterns align with the 5′-G-Py*−3’ rule, which describes how bleomycin can specifically bind to 5′-G-T*/C*−3′ and induce mutations at the star-marked pyrimidine (18, 19). In summary, our analysis reveals that different mutagenic conditions lead to specific sequence context biases around SNV sites, highlighting the context-dependent interactions of various mutagens with DNA.

### Zeocin induces more frequent InDels in the *Y. lipolytica* genome than UV and MMS

InDels were categorized into three classes: 1, 2, and ≥3 bp deletions or insertions. We observed that 1 bp deletions occurred at a much higher rate (8.36 × 10⁻^11^/base/division, 95% CI: 5.47–10.83 × 10⁻^11^) compared to other types of spontaneous InDels (Table 2). UV treatment significantly increased the rates of all types of InDels, while MMS specifically raised the rate of 1 bp insertions by threefold (Table 2). In Zeocin-treated cells, ≥3 bp deletions were the most frequent, followed by 1 bp deletions (Table S3). Compared to untreated cells, ≥3 bp deletions were elevated by 189-fold, and 1 bp deletions increased by 18-fold in Zeocin-treated cells (Table 2). Our data confirmed that UV, MMS, and Zeocin each exert distinct effects on the frequency and ratios of various types of InDels in the *Y. lipolytica* genome, with Zeocin exhibiting the highest induction capability. The reasons for the increased frequency of ≥3 and 1 bp deletions compared to other InDels will be discussed in the Discussion section.

**TABLE 2:** Rates of InDels in *Y. lipolytica* under spontaneous and mutagen-induced conditions

InDels	Absolute mutation rate (×10^−11^)			
	Spontaneous^*a*^	Zeocin	MMS^*b*^	UV
1 bp deletions	8.36 (5.47–10.83)	149.54 (99.20–194.12)	11.58 (0–24.17)	29.08 (9.67–37.81)
1 bp insertions	4.27 (2.25–6.06)	36.78 (13.05–52.86)	11.58 (0–30.15)	20.64 (9.20–32.42)
2 bp deletions	0.59 (0.13–1.03)	27.99 (13.39–41.28)	NA	4.69 (0–7.78)
2 bp insertions	1.25 (0.51–1.85)	7.20 (0–13.61)	NA	5.63 (0–17.59)
≥3 bp deletions	0.92 (0.23–1.36)	173.53 (116.82–218.44)	NA	5.63 (0–10.92)
≥3 bp insertions	0.67 (0.20–1.11)	5.60 (0–9.11)	NA	1.88 (0–3.44)

^*a*^ The values in parentheses indicate the 95% CI. The unit of the rates was per base per cell division.

^*b*^ NA indicates no events were detected.

### NHEJ and translesion synthesis pathways contribute to Zeocin-induced point mutations

**Fig 4:**
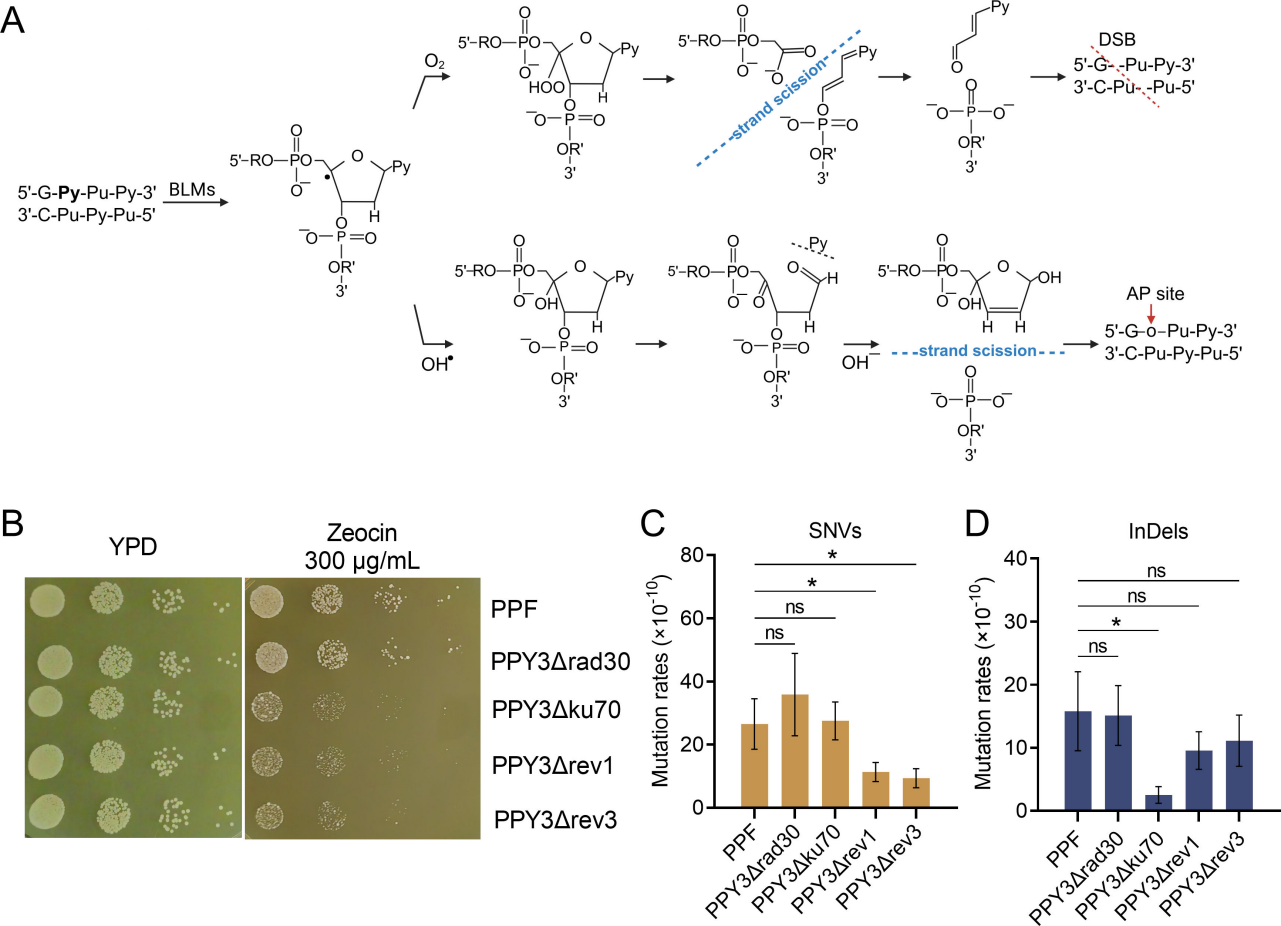
The role of NHEJ and translesion synthesis (TLS) pathway mutations. (**A**) Zeocin-induced DNA lesions, primarily including abasic (AP) sites and DNA breaks. (**B**) Effects of deleting *RAD30* (PPY3Δrad30), *REV1* (PPY3Δrev1), *REV3* (PPY3Δrev3), and *KU70* (PPY3Δku70) on Zeocin resistance. (**C**) Comparison of SNV rates between the reference strain (PPF) and the TLS and NHEJ mutants. (**D**) Rates of InDels in the PPF strain compared to the TLS and NHEJ mutants.

Zeocin, a member of the bleomycin family, can remove the 4′-hydrogen atom from the C4′ position of the deoxyribose moiety of pyrimidines, generating 4′-radical intermediates that can subsequently be converted into 4′-oxidized apurinic/apyrimidinic (AP) sites or gapped DNA with 3′-phosphoglycolate/5′-phosphate ends (Fig. 4A) (37, 38). Incision of the opposite strand leads to double-strand breaks (DSBs) (Fig. 4A). NHEJ, which is preferred over homologous recombination in *Y. lipolytica* (39, 40), is the most likely mechanism for repairing Zeocin-induced DSBs. To assess the role of NHEJ in Zeocin-induced mutations, we deleted the Ku70-encoding gene *YALI1_C11925g* in strain PPY3 to construct an NHEJ-deficient mutant, PPY3Δku70 (Table 3). This mutant displayed increased sensitivity to Zeocin compared to PPF (Fig. 4B), highlighting the importance of NHEJ in repairing Zeocin-induced DNA damage. We subcultured 10 PPY3Δku70-derived isolates and 10 PPF-derived isolates on YPD plates containing 60 µg/mL Zeocin for 10 generations, followed by sequencing. Our results indicated that the rates of SNVs and InDels in the *ku70* mutant were 2.8 × 10^−9^ and 3 × 10^−10^/base/cell division, respectively, suggesting that approximately 80% of the Zeocin-induced InDels rely on the NHEJ pathway (Fig. 4C). This finding is consistent with the fact that Zeocin is a potent mutagen causing DSBs. However, the rate of Zeocin-induced SNVs was not significantly affected by the absence of Ku70.

**TABLE 3:** *Y. lipolitica* strains used in this study

Strain	Genotype	Source
W29	*MatA*	(11)
PO1f	*MatA leu2-270 ura3-302 xpr2-322 axp1-2*	(41)
PPF	*MatA leu2-270 ura3-302 xpr2-322 axp1-2 int::CAS9-LEU2*	This work
PPY3	*MatA leu2-270 ura3-302 xpr2-32 axp1-2 int::CAS9-LEU2 LYS9-Δ*	This work
PPY3Δku70	*MatA leu2-270 ura3-302 xpr2-322 axp1-2 int::CAS9-LEU2 LYS9-Δ KU70Δ*∷*LYS9*	This work
PPY3Δrev1	*MatA leu2-270 ura3-302 xpr2-322 axp1-2 int::CAS9-LEU2 LYS9-Δ REV1Δ*∷*LYS9*	This work
PPY3Δrev3	*MatA leu2-270 ura3-302 xpr2-322 axp1-2 int::CAS9-LEU2 LYS9-Δ REV3Δ∷LYS9*	This work
PPY3Δrad30	*MatA leu2-270 ura3-302 xpr2-322 axp1-2 int::CAS9-LEU2 LYS9-Δ RAD30Δ∷LYS9*	This work
PPW	*MatA leu2-270 ura3-302 xpr2-322 axp1-2 int::CAS9-LEU2 LYS9-Δ int∷LYS9 int∷URA3*	This work
PPY3ΔE14028g	*MatA leu2-270 ura3-302 xpr2-322 axp1-2 int::CAS9-LEU2 LYS9-Δ YALI1_E14028gΔ::LYS9 int::URA3*	This work
PPY3ΔF15859g	*MatA leu2-270 ura3-302 xpr2-322 axp1-2 int::CAS9-LEU2 LYS9-Δ YALI1_F15859gΔ::LYS9 int::URA3*	This work
PPY3ΔA00538g	*MatA leu2-270 ura3-302 xpr2-322 axp1-2 int::CAS9-LEU2 LYS9-Δ YALI1_A00538gΔ::LYS9 int::URA3*	This work
PPY3ΔE21053g	*MatA leu2-270 ura3-302 xpr2-322 axp1-2 int::CAS9-LEU2 LYS9-Δ YALI1_E21053gΔ::LYS9 int::URA3*	This work
PPY3ΔB18292g	*MatA leu2-270 ura3-302 xpr2-322 axp1-2 int::CAS9-LEU2 LYS9-Δ YALI1_B18292gΔ::LYS9 int::URA3*	This work
PPY3ΔB25553g	*MatA leu2-270 ura3-302 xpr2-322 axp1-2 int::CAS9-LEU2 LYS9-Δ YALI1_B25553gΔ::LYS9 int::URA3*	This work
PPY3ΔF26427g	*MatA leu2-270 ura3-302 xpr2-322 axp1-2 int::CAS9-LEU2 LYS9-Δ YALI1_F26427gΔ::LYS9 int::URA3*	This work
PPY3ΔC11234g	*MatA leu2-270 ura3-302 xpr2-322 axp1-2 int::CAS9-LEU2 LYS9-Δ YALI1_C11234gΔ::LYS9 int::URA3*	This work

AP sites, one of the most common DNA lesions in Zeocin-treated cells, hinder DNA replication because the primary replicative polymerases are inefficient at inserting correct nucleotides opposite the AP site (42). In eukaryotes, certain TLS polymerases can bypass AP sites through error-prone nucleotide incorporation (43). While these TLS polymerases enable cells to replicate damaged DNA, they do so at the expense of increased mutations. To assess the role of the TLS pathway in Zeocin-induced mutations in *Y. lipolytica*, we knocked out the genes *RAD30*, *REV1*, and *REV3*, which encode polymerases η, Rev1, and ζ, respectively, in strain PPY3. The resulting mutants were designated PPY3Δrad30, PPY3Δrev1, and PPY3Δrev3. These three mutants displayed growth rates comparable to that of the parental strain; however, PPY3Δrev1 and PPY3Δrev3 formed smaller colonies than PPF on plates containing Zeocin, while PPY3Δrad30 did not show any reduction in colony size (Fig. 4B). This result suggests that DNA polymerases Rev1 and ζ are essential for repairing Zeocin-induced DNA lesions and thus contribute to Zeocin resistance in *Y. lipolytica*. Sequencing of Zeocin-treated isolates (subcultured on Zeocin plates as PPF and PPY3Δku70) from these TLS-deficient mutants revealed that the SNV rates in PPY3Δrev1 (11.3 × 10^−10^/base/cell division) and PPY3Δrev3 (9.4 × 10^−10^/base/cell division) were 59% and 67% lower, respectively, compared to the reference strain PPF. Detailed information on the SNVs and InDels is listed in Data set S8 to S10. We also observed that InDels rates in the *rev1* and *rev3* mutants were reduced by 40% and 30%, respectively, though these differences were not statistically significant at the 0.05 level (Fig. 4D, Wilcoxon rank-sum test). Overall, these results suggest that Zeocin-induced SNVs in *Y. lipolytica* largely depend on the polymerases Rev1 and ζ-mediated TLS pathways. Additionally, these two TLS polymerases may, at least in part, contribute to the Zeocin-induced InDels.

### Zeocin-induced InDels conferred Zeocin tolerance to *Y. lipolytica*

**Fig 5:**
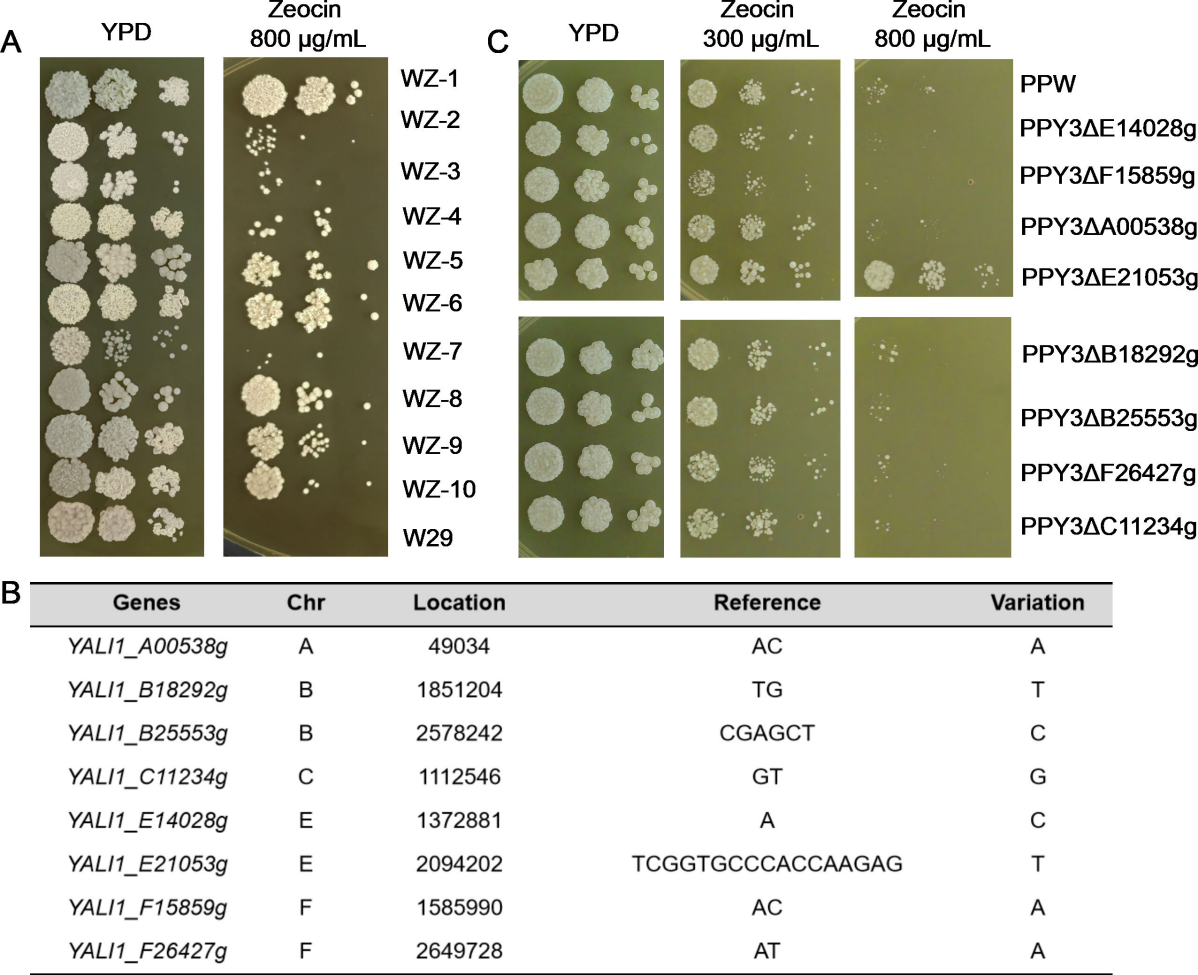
Increased Zeocin resistance of subcultured isolates and underlying mechanisms in *Y. lipolytica*. (**A**) All WZ strains showed enhanced tolerance to Zeocin. Ten mutant strains (WZ1~WZ10) and the parental strain W29 were separately spotted on YPD and 800 µg/mL Zeocin-containing plates and then incubated at 30°C for 2 days. (**B**) Eight genes potentially associated with Zeocin tolerance. (**C**) Comparative growth of PPW and the gene deletion mutant on YPD and Zeocin plates. The deletion of gene *YALI1_E21053g* led to a higher Zeocin resistance.

Considering our results, which confirm that UV, MMS, and Zeocin can effectively mutagenize *Y. lipolytica* genome, we investigated whether the induced mutations contribute to the phenotypic evolution. Spotting assay demonstrated that several subcultured isolates were more resistant to UV or MMS (Fig. S3), while all 10 isolates (WZ1–10) subcultured on Zeocin-containing plates exhibited greater tolerance than the parent strain W29 (Fig. 5A). This result suggested that *Y. lipolytica* strains can readily acquire Zeocin tolerance during the subculture process. Compared to UV and MMS, Zeocin is more likely to cause InDels that might be responsible for the improved Zeocin tolerance. Since open reading frame (ORF) shifts can disrupt protein function, we assessed the phenotypic effects of frameshift-induced InDels by deleting entire ORFs. Among 501 InDels in the genomes of WZ isolates, 91 caused frameshifts in genes (Data set S5). We randomly deleted seven candidate genes with frameshifting InDels and one candidate gene with missense in the *Y. lipolytica* PPW strain to evaluate their effects on Zeocin resistance (Fig. 5B). Our results showed that only the *YALI1_E21053g* null mutant grew better than the reference strain PPW on Zeocin-containing medium (Fig. 5C).

To further identify mutations selected under Zeocin stress during the early phase of MAE, we subcultured eight independent isolates in the presence of Zeocin for only two cycles to minimize mutation accumulation. Among the 8 sequenced isolates, we detected 50 SNVs and 37 InDels (Data set S11), with rates of 6.1 × 10^−9^ and 4.5 × 10^−9^/base/cell division, respectively. Of the 37 InDels, 8 caused frameshifts, and 3 of these frameshift-inducing InDels occurred in the gene *YALI1_E21053g* across three independent isolates (Data set S11). These findings suggest that frameshift-induced deactivation of *YALI1_E21053g* is a preferential mechanism for enhancing Zeocin resistance compared to other mutations. In summary, our results indicate that Zeocin’s strong InDels-inducing capability may be a valuable tool for developing microorganisms with specific desired traits. The potential physiological mechanisms of Zeocin resistance conferred by the absence of *YALI1_E21053g* are discussed below.

## DISCUSSION

This study elucidated the patterns of spontaneous and mutagen-induced genomic alterations in *Y. lipolytica*. Our key findings are summarized as follows: (i) spontaneous SNVs and InDels occurred at rates of 2.07 (95% CI: 1.71–2.43) × 10^−10^ and 1.61 (95% CI: 1.11–2.01) × 10^−10^/base/cell division, respectively; among the SNVs, C-to-T/G-to-A and C-to-A/G-to-T substitutions were most common, while single-base deletions were the predominant type of InDel; (ii) chromosomal rearrangements and aneuploidy were relatively infrequent in *Y. lipolytica* under spontaneous conditions; (iii) treatments with UV, MMS, and Zeocin resulted in distinctly different spectra and distributions of SNVs and InDels; (iv) NHEJ and TLS were the primary pathways responsible for Zeocin-induced point mutations, which preferentially occurred at 5′-TGPy-3′ motifs; and (v) *Y. lipolytica* cells could rapidly acquire Zeocin resistance, attributed to the high frequency of Zeocin-induced InDels. The implications of these findings will be discussed below.

### Patterns of spontaneous genomic alterations of *Y. lipolytica*

**Fig 6:**
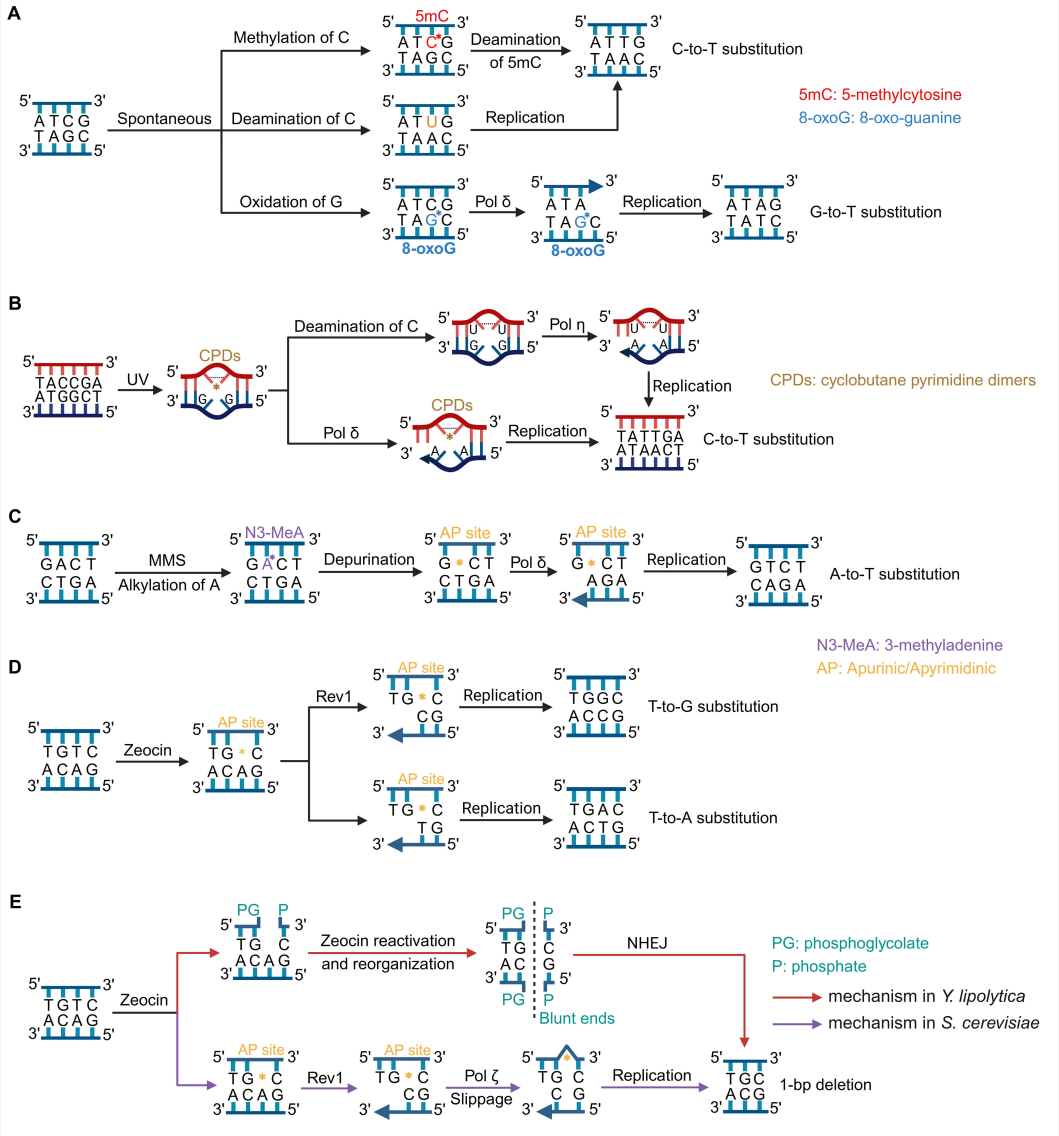
Models of signature mutations of *Y. lipolytica* under various conditions. (**A**) C-to-T and G-to-T substitution under spontaneous conditions. (**B**) UV induced C-to-T was associated with cyclobutane pyrimidine dimers (CPDs) formation. (**C**) MMS-induced A–T was presumed to be caused by 3-methyladenine (N3-MeA). (**D**) Zeocin-induced T–A and T–G were dependent on AP site formation and translesion synthesis. (**E**) Two distinct mechanisms underlying Zeocin-induced 1 bp deletion.

Our results revealed that a single base in the *Y. lipolytica* genome was mutated approximately once every 236 cell divisions under spontaneous conditions, similar to observations in *S. cerevisiae* (33, 44). The most common spontaneous SNVs in both yeasts were C-to-T/G-to-A transitions. These predominant substitutions are likely due to DNA methylation and spontaneous base deamination (45) (Fig. 6A). Specifically, C can be methylated by DNA methyltransferase and S-adenosylmethionine to form 5-methylcytosine (5mC). The deamination of 5mC, involving the loss of its exocyclic amine group, converts it to T (Fig. 6A). Additionally, cytosine can lose its exocyclic amine to become uracil (U), which pairs with A, leading to C-to-T substitutions in the subsequent round of DNA replication (Fig. 6A). The second most common SNVs in the *Y. lipolytica* genome were C-to-A/G-to-T transversions, likely resulting from the oxidation of guanine to 8-oxo-guanine, which preferentially pairs with adenine (Fig. 6A) (46). In both haploid and diploid *S. cerevisiae*, approximately 74% of spontaneous SNVs occur within coding regions, which closely aligns with the expected ratio of coding regions (73.9%) (33). Conversely, the genome of *Y. lipolytica* consists of 50.7% non-coding regions, a much larger proportion compared to *S. cerevisiae*. In our study, out of 247 spontaneous SNVs identified, 156 were found in non-coding regions, showing a higher mutation rate in these areas for *Y. lipolytica* (chi-squared test, *P* < 0.01). We also observed that the ratio of intergenic to intragenic SNVs in *Y. lipolytica* varied across different treatment conditions (Fig. S2). These findings suggest that both the rate and distribution of SNVs in *Y. lipolytica* are influenced by various growing conditions.

Contrary to our expectations, the spontaneous rate of InDels in *Y. lipolytica* (1.61 × 10^−10^/base/cell division, 95% CI: 1.11–2.01 × 10⁻^10^) was found to be approximately an order of magnitude higher than that in *S. cerevisiae* (33). The majority of these spontaneous InDels in *Y. lipolytica* were identified as 1 bp deletions, with 91% occurring within mononucleotide tandem repeat sequences (Data set S2). This finding suggests that template slippage errors, which escape DNA repair, are the most likely cause of these spontaneous InDels. To investigate further, we calculated the total number and length of mononucleotide repeats in both *Y. lipolytica* and *S. cerevisiae* (Table S3). Our analysis showed that *Y. lipolytica* has a relatively lower ratio of mononucleotide repeats compared to *S. cerevisiae* (Table S3). This suggests that the elevated rate of 1 bp deletions in *Y. lipolytica* is not attributable to a greater abundance of mononucleotide repeats in its genome. Given that the mismatch repair pathway is the primary mechanism for correcting errors arising from template slippage (47), one possible explanation for the higher InDels rate observed in *Y. lipolytica* is that this pathway may be less efficient in *Y. lipolytica* compared to *S. cerevisiae*.

Chromosomal rearrangements and aneuploidy events occur occasionally in the *Y. lipolytica* genome, with rates of 5.14 × 10⁻⁵ (95% CI: 0–11.80 × 10^−^⁵) and 1.71 × 10⁻⁵ events per cell division, respectively. While it is not possible to directly compare these rates to those of haploid *S. cerevisiae* due to a lack of available data, these events have been observed at higher rates in diploid *S. cerevisiae*, approximately 1.8 × 10⁻⁴ and 6.4 × 10⁻⁵ events per cell division, respectively (31). Transposon elements are often found at the breakpoints of chromosomal rearrangements in *S. cerevisiae*, indicating that long repeats can mediate unequal crossover between non-allelic loci (48). However, this phenomenon was not observed in the chromosomal rearrangements detected in *Y. lipolytica* in this study, which aligns with the fact that this species exhibits weak homologous recombination capabilities (39). Additionally, it is important to note that selection pressure is more effective against large-scale chromosomal aberrations in haploid organisms, including *Y. lipolytica*, as large deletions and chromosomal losses are often lethal in haploids.

### Different mutagens led to distinct mutation spectrum in *Y. lipolytica*

A key finding of this study is the identification of distinct mutation signatures associated with each mutagen. Similar to observations in other organisms (13, 49, 50), UV light serves as a potent mutagen in *Y. lipolytica*. Under UV exposure, the predominant SNVs identified were C-to-T substitutions (Fig. 2B and 6B). Additionally, these SNVs frequently occurred with T or C at the 5’ position, consistent with the fact that the main DNA lesions caused by UV exposure are cyclobutane pyrimidine dimers (CPDs) (51). Previous studies in *S. cerevisiae* have also highlighted the mutagenic potential of pyrimidine dimers (52, 53). CPDs present significant obstacles to replicative polymerases, necessitating the involvement of TLS polymerases to bypass these lesions, albeit with an increased risk of introducing mutations (51, 54). It has been reported that CPDs serve as templates for DNA polymerase η, which can facilitate the deamination of cytosine (C to U) within the dimers. This process facilitates the insertion of A opposite the deaminated sites, leading to C-to-T transitions (Fig. 6B) (55). However, we did not rule out the possibility that other polymerases, including DNA polymerase δ, might also be involved in the TLS of CPDs (Fig. 6B) (56, 57). In conclusion, the UV-induced C-to-T substitutions observed in this study are likely a result of TLS-dependent bypassing of CPDs.

MMS is a potent alkylating agent that primarily induces DNA damage by adding alkyl groups to DNA bases. The most common lesions caused by MMS are the alkylation of the N7 position of guanine and the N3 position of adenine, leading to the formation of 7-methylguanine (N7-MeG) and 3-methyladenine (N3-MeA), respectively (58). These modified bases are particularly prone to depurination, resulting in the formation of AP sites (Fig. 6C). In this study, we demonstrated that A-to-T mutations were predominantly induced in MMS-treated *Y. lipolytica* cells. One plausible explanation for this mutation signature is that DNA polymerase δ often incorporates A opposite the AP sites formed by N3-MeA, frequently leading to A-to-T substitutions (Fig. 6C). In addition to N7-MeG and N3-MeA, MMS can also generate minor alkyl DNA adducts at other positions, such as the O6 atom of guanine. The formation of O6-methylguanine is particularly mutagenic, as it can mispair with T, leading to G-to-A transitions (59, 60). This mechanism may partially explain the significant proportion of G-to-A mutations (33% of all SNVs) observed in MMS-treated *Y. lipolytica*. An intriguing finding in this study was the strong sequence context dependency of MMS-induced A-to-C substitutions, with the prominent motif being 5′-CA*−3′. The exact mechanism underlying this sequence bias remains to be explored in future research.

In Zeocin-treated *Y. lipolytica* isolates, SNVs predominantly occurred at thymine bases, consistent with observations in *S. cerevisiae* (18). However, the relative ratios of base substitutions differed between the two species. In *S. cerevisiae*, the most common SNVs at thymine bases are T-to-G substitutions, accounting for up to 43% of all SNVs (61, 62). This pattern is likely due to the activity of Rev1, which preferentially incorporates C opposite AP sites, followed by extension by DNA polymerase ζ (Fig. 6D) (61, 62). In this study, we found that Rev1 and polymerase ζ-mediated TLS are also crucial for the survival of *Y. lipolytica* cells exposed to Zeocin-induced DNA lesions. Mutants lacking Rev1 or Rev3 showed increased sensitivity to Zeocin, and the SNV rates decreased by 59% and 67%, respectively (Fig. 4B and C). In contrast, the absence of DNA polymerase η (Rad30) did not significantly affect Zeocin resistance or the mutation rate (Fig. 4B through D). Interestingly, in Zeocin-treated *Y. lipolytica* isolates, T-to-A substitutions occurred at a 2.1-fold higher rate than T-to-G substitutions, although both substitutions followed the 5′-GT*−3′ rule (63–67). This discrepancy may be due to a unique preference in *Y. lipolytica* Rev1 for incorporating T rather than cytosine opposite AP sites. Alternatively, uncertain DNA polymerases may be responsible for inserting T at these sites, while the subsequent extension requires Rev1 and polymerase ζ (Fig. 6D). Overall, these findings highlight the significant role of the Rev1 and polymerase ζ pathway in mediating DNA damage responses in *Y. lipolytica*, with distinct mutation signatures that differ from those observed in *S. cerevisiae*, reflecting species-specific variations in TLS mechanisms.

### Mechanisms underlying Zeocin-induced InDels

Zeocin treatment resulted in a significant increase in InDels, with a notable prevalence of 1 bp (37%) and ≥3 bp (43%) deletions. Frequent 1 bp deletions have been reported in *S. cerevisiae* and higher eukaryotes treated with bleomycin (18, 68, 69). For instance, in Chinese Hamster Ovary D422 cells treated with bleomycin, 1 bp deletions constituted nearly half of all mutations (68). The deletion of the *KU70* gene in *Y. lipolytica* led to a significant decrease in InDels rates (Fig. 4D), confirming the role of the NHEJ pathway in repairing Zeocin-induced DSBs and facilitating InDel formation (Fig. 6E). Bleomycin is known to create blunt-ended DSBs, and the NHEJ pathway’s repair of these breaks can result in 1 bp deletions (Fig. 6E) (65, 70–73). The ≥3 bp deletions observed are likely indicative of a process where the excision of “sticking” bases at the break ends occurs to facilitate end joining. Also, adjacent 5′-GT*−3′ motifs may increase the likelihood of generating closely spaced DSBs and lead to deletions of 3 bp or more following end joining by NHEJ.

In contrast, the deletion of *KU70* did not significantly affect Zeocin resistance or InDels rates in either haploid or diploid *S. cerevisiae* strains (18). The authors of the referenced studies proposed a template slippage model to explain the 1 bp deletions observed in *S. cerevisiae*: Rev1 inserts C opposite an AP site at the 5′-GT-3′ motif, which can easily pair with the G base located 5′ to the AP site due to template slippage (Fig. 6E) (18, 74). DNA polymerase ζ then extends from this inserted nucleotide, resulting in a 1 bp deletion at the original site (74–77). Although the results were not statistically significant at the 0.05 level, we observed that the deletion of *REV1* and *REV3* also led to a reduction in InDels in *Y. lipolytica* (Fig. 4D). This finding suggests that the TLS pathway may contribute to 1 bp deletions in Zeocin-treated cells, albeit to a much lesser extent compared to *S. cerevisiae*. Indeed, the lower frequency of T-to-G substitutions at the 5′-GT*−3′ motif in *Y. lipolytica* compared to *S. cerevisiae* suggests that either fewer cytosines were incorporated opposite the AP sites or that fewer AP sites were formed within this sequence context. This reduction would limit the occurrence of template slippage and, consequently, the formation of 1 bp deletions in *Y. lipolytica*.

In summary, our findings underscore the critical but distinct roles of the NHEJ and TLS pathways in InDel formation following Zeocin treatment, with species-specific mechanisms contributing to the observed mutation patterns, highlighting the importance of understanding *in vivo* functions of these pathways in different organisms.

### Zeocin exposure readily evolves Zeocin tolerance

In this study, all subcultured isolates exhibited increased tolerance to Zeocin compared to the parental strain (Fig. 5A), a phenomenon that mirrors observations in cancer cell lines where long-term exposure to bleomycin leads to the development of bleomycin resistance (78). Through the annotation of mutations in Zeocin-treated isolates and subsequent validation via genetic manipulation, we demonstrated that the inactivation of the gene *YALI1_E21053g* leads to increased Zeocin resistance. *YALI1_E21053g* encodes a serine/threonine kinase with homology to the *PTK2* gene in *S. cerevisiae*. It has been established that Ptk2 positively regulates membrane transport, including the import of extracellular polyamines (79, 80). In *S. cerevisiae*, deletion of *PTK2* resulted in enhanced bleomycin tolerance due to the deactivation of the bleomycin transporter Agp2 (79). Thus, it is reasonable to hypothesize that the frameshift InDels in *YALI1_E21053g* likely reduce the import of Zeocin into *Y. lipolytica* cells, contributing to improved growth in the presence of the drug. These findings suggest that frameshift-induced deactivation of *YALI1_E21053g* is a preferential mechanism for enhancing Zeocin resistance compared to other mutations.

### Conclusion

This study revealed the distinct patterns of genomic alterations that arise under both spontaneous and mutagen-induced conditions. The results suggest that combining multiple mutagens can enhance the diversity and distribution of mutations, which could be highly beneficial for developing complex traits in genetic breeding programs. Importantly, because *Y. lipolytica* shares more genomic and DNA repair pathway similarities with metazoans than *S. cerevisiae*, studying genomic alterations in this organism could provide valuable insights that are more directly applicable to higher eukaryotes. This research underscores the potential of *Y. lipolytica* as a model organism for studying genomic alterations and its implications for broader applications in genetics and biotechnology.

## MATERIALS AND METHODS

### Strains and media

The wild-type *Y. lipolytica* strain W29 (*MatA*, ATCC 20460) served as the parent strain for the MAE experiment (11). For genetic manipulation, we used the W29-derived strain PO1f (*MatA leu2-270 ura3-302 xpr2-322 axp1-2*) (41). To facilitate gene knockout, we integrated a *CAS9* gene into the PO1f genome, generating the mutant strain PPF (*MatA leu2-270 ura3-302 xpr2-322 axp1-2 int::CAS9-LEU2*) (Table 3). The knockout of *LYS9* in PPF resulted in strain PPY3 (*MatA leu2-270 ura3-302 xpr2-322 axp1-2 int::CAS9-LEU2 LYS9-Δ*). The insertion of *LYS9* and *URA3* genes in the genome PPY3 resulted in strain PPW (*MatA leu2-270 ura3-302 xpr2-322 axp1-2 int::CAS9-LEU2 LYS9-Δ int∷LYS9 int∷URA3*). This strain served as a control for evaluating the phenotypic effects of gene deletions. The genotypes of the *Y. lipolytica* strains and the primers used for strain construction are detailed in Table 1; Table S1, respectively. The detailed processes of strain construction are shown in the supplemental text (Supplementary file S1). Yeast transformations were performed using the LiAc/SS-DNA/PEG protocol (81).

*Y. lipolytica* was cultured in YPD medium, which contains 20 g/L glucose (SCR, Shanghai, China), 20 g/L tryptone (Oxoid, Basingstoke, UK), and 10 g/L yeast extract (Oxoid). The nutritional deficiency screening medium was prepared with 6.7 g/L yeast nitrogen base (Coolaber, Beijing, China), 20 g/L glucose, and 1.4 g/L drop-out supplement (Aladdin, Shanghai, China), with 20 g/L agar (SCR) added to make solid medium.

### MAE experiments

*Y. lipolytica* W29 cells were plated on solid YPD plates and incubated at 30°C for 2 days. Twenty-one colonies were randomly selected as parental isolates and independently streaked to single cells on new YPD plates. After 2 days of incubation, one colony from each isolate was picked and streaked onto fresh YPD plates. This process was repeated for either 103 cycles (11 isolates) or 120 cycles (10 isolates) to accumulate mutations. For mutagenesis, MMS and Zeocin were added to the YPD medium at final concentrations of 0.03% and 60 µg/mL, respectively, allowing approximately 65% of the cells to form colonies. Eight independent isolates of W29 were subcultured on 0.3% MMS-containing plates at 30°C for either 13 cycles (six isolates) or 20 cycles (two isolates). Eight were subcultured on 60 µg/mL Zeocin-containing plates at 30°C for either 22 cycles (four isolates) or 39 cycles (four isolates). For UV treatment, nine isolates were subcultured on YPD plates under conditions identical to the spontaneous treatment for either 16 cycles (five isolates) or 32 cycles (four isolates), but the cells were exposed to 80 J/m² UV after each streaking cycle, resulting in approximately 40% loss of cell viability.

### Whole-genome sequencing and data analysis

Yeast cells were incubated in 15 mL of YPD medium for 20 hours, and approximately 5 × 10^−8^ cells were then collected by centrifugation (6,000 rpm for 5 minutes) for genomic DNA extraction using the EZNA Yeast DNA Kit (Omega, Doraville, GA). The genomic DNA was sheared by sonication to fragments of approximately 400 bp for sequencing library construction using a commercial kit (specific kit name not provided). Sequencing was performed on the BGI MGISEQ-2000 platform using a 2 × 150 bp paired-end protocol.

Clear reads (approximately 2 G for each sample) were mapped onto the *Y. lipolytica* genome (https://www.ncbi.nlm.nih.gov/datasets/genome/GCA_001761485.1/) using the bwa-mem algorithm of the software BWA (82). Single-nucleotide variants and insertions or deletions were detected using the software SAMtools (83) and VarScan (84), as described in our previous studies (21, 85). Genome assembly was conducted using SPAdes (86), and genome alignment was performed with BLAT (87).

Mutation rate $\mu_{bp}$ per base pair per generation was calculated as follows: $\mu_{bp}=\frac{n_{bp}}{N\times gen_{tot}\times t}$, where *n*_bp_ is the number of mutations of any type of SNVs or InDels; *N* is 20,500,000, the number of base pairs of *Y. lipolytica* genome; gen_tot_ is the total number of subculture generations; and *t* is the division numbers from a single cell to a colony. Mutation rates per arrangement or aneuploidy per cell division ($\mu_{lar}$) were calculated using the formula: $\mu_{lar}=\frac{n_{lar}}{{gen}_{tot}\times t}$, where *n*_lar_ is the number of arrangements, with gen_tot_ and *t* defined as above. Mutation rates and ratios were plotted using GraphPad Prism 9.5.0 (88).

### Mutational signature analysis

To generate mutation signatures, we extracted trinucleotide sequences from both the reference genome and the sequencing data, positioning the mutated base in the center of each sequence. The trinucleotide sequences were classified into 96 different categories based on six substitution types and the combinations of two nucleotides flanking the mutated pyrimidines. This classification was used to create single-base substitution signatures, which are available at COSMIC (https://cancer.sanger.ac.uk/cosmic/signatures/SBS). Sequence logos for base substitutions were generated using WebLogo 3 (https://weblogo. threeplusone.com/create.cgi) (89).

### Plasmid construction

The pCRISPRyl was purchased from Addgene (https://www.addgene.org/70007/) (90). Using this plasmid as a PCR template, we obtained elements of selection marker *AmpR*, *SCR1-tRNA^Gly^* promoter, gRNA scaffold, and *CEN* and *ARS* (Fig. S1). A *URA3* gene was amplified from the genomic DNA of strain W29 by PCR. These elements were assembled to generate the plasmid pU2gR that is able to express two sgRNAs (Fig. S1). The sgRNA sequences for gene knockout was designed using CRISPOR (http://crispor.tefor.net/) (Table S1).

## Data Availability

The raw sequencing data are available in the NCBI Sequence Read Archive database under accession number PRJNA1150590.

## SUPPLEMENTAL MATERIAL

Data Sets S1 to S11 aem.01678-24-s0001.xlsx WGS data of mutants.

Supplemental material aem.01678-24-s0002.docx Tables S1 to S3; Fig. S1 to S3.

ASM does not own the copyrights to Supplemental Material that may be linked to, or accessed through, an article. The authors have granted ASM a non-exclusive, world-wide license to publish the Supplemental Material files. Please contact the corresponding author directly for reuse.

## References

[1] Dujon B, Sherman D, Fischer G, Durrens P, Casaregola S, Lafontaine I, de Montigny J, Marck C, Neuvéglise C, Talla E, et al.. 2004. Genome evolution in yeasts. Nat New Biol 430:35–44. doi:10.1038/nature02579 15229592

[2] Choi S, Song CW, Shin JH, Lee SY. 2015. Biorefineries for the production of top building block chemicals and their derivatives. Metab Eng 28:223–239. doi:10.1016/j.ymben.2014.12.007 25576747

[3] Lian J, Mishra S, Zhao H. 2018. Recent advances in metabolic engineering of Saccharomyces cerevisiae: new tools and their applications. Metab Eng 50:85–108. doi:10.1016/j.ymben.2018.04.011 29702275

[4] Spohner SC, Schaum V, Quitmann H, Czermak P. 2016. Kluyveromyces lactis: an emerging tool in biotechnology. J Biotechnol 222:104–116. doi:10.1016/j.jbiotec.2016.02.023 26912289

[5] Baghban R, Farajnia S, Rajabibazl M, Ghasemi Y, Mafi A, Hoseinpoor R, Rahbarnia L, Aria M. 2019. Yeast expression systems: overview and recent advances. Mol Biotechnol 61:365–384. doi:10.1007/s12033-019-00164-8 30805909

[6] Abdel-Mawgoud AM, Markham KA, Palmer CM, Liu N, Stephanopoulos G, Alper HS. 2018. Metabolic engineering in the host Yarrowia lipolytica. Metab Eng 50:192–208. doi:10.1016/j.ymben.2018.07.016 30056205

[7] Miller KK, Alper HS. 2019. Yarrowia lipolytica: more than an oleaginous workhorse. Appl Microbiol Biotechnol 103:9251–9262. doi:10.1007/s00253-019-10200-x 31686142

[8] Liu HH, Ji XJ, Huang H. 2015. Biotechnological applications of Yarrowia lipolytica: past, present and future. Biotechnol Adv 33:1522–1546. doi:10.1016/j.biotechadv.2015.07.010 26248319

[9] Bankar A, Zinjarde S, Shinde M, Gopalghare G, Ravikumar A. 2018. Heavy metal tolerance in marine strains of Yarrowia lipolytica. Extremophiles 22:617–628. doi:10.1007/s00792-018-1022-y 29594464

[10] Bankar AV, Kumar AR, Zinjarde SS. 2009. Environmental and industrial applications of Yarrowia lipolytica. Appl Microbiol Biotechnol 84:847–865. doi:10.1007/s00253-009-2156-8 19669134

[11] Madzak C. 2021. Yarrowia lipolytica strains and their biotechnological applications: how natural biodiversity and metabolic engineering could contribute to cell factories improvement. J Fungi (Basel) 7:548. doi:10.3390/jof7070548 34356927 PMC8307478

[12] Magnan CN, Yu J, Chang I, Jahn E, Kanomata Y, Wu J, Zeller MD, Oakes ML, Baldi P, Sandmeyer SB. 2016. Sequence assembly of Yarrowia lipolytica strain W29/CLIB89 shows transposable element diversity. PLoS ONE 11:e0162363. doi:10.1371/journal.pone.0162363 27603307 PMC5014426

[13] Yin Y, Petes TD. 2013. Genome-wide high-resolution mapping of UV-induced mitotic recombination events in Saccharomyces cerevisiae. PLoS Genet 9:e1003894. doi:10.1371/journal.pgen.1003894 24204306 PMC3814309

[14] Barbour L, Hanna M, Xiao W. 2006. Mutagenesis. Methods Mol Biol 313:121–127. doi:10.1385/1-59259-958-3:121 16118430

[15] Sugiyama T, Keinard B, Best G, Sanyal MR. 2021. Biochemical and photochemical mechanisms that produce different UV-induced mutation spectra. Mutat Res 823:111762. doi:10.1016/j.mrfmmm.2021.111762 34563793 PMC8671204

[16] Maxwell PH. 2016. Growth conditions that increase or decrease lifespan in Saccharomyces cerevisiae lead to corresponding decreases or increases in rates of interstitial deletions and non-reciprocal translocations. BMC Genet 17:140. doi:10.1186/s12863-016-0447-5 27769161 PMC5073950

[17] Lee GS, Blonsky KS, Van On DL, Savage EA, Morgan AR, von Borstel RC. 1992. Base alterations in yeast induced by alkylating agents with differing Swain-Scott substrate constants. J Mol Biol 223:617–626. doi:10.1016/0022-2836(92)90978-s 1542109

[18] Zheng DQ, Wang YT, Zhu YX, Sheng H, Li KJ, Sui Y, Zhang K. 2022. Uncovering bleomycin-induced genomic alterations and underlying mechanisms in the yeast Saccharomyces cerevisiae. Appl Environ Microbiol 88:e0170321. doi:10.1128/AEM.01703-21 34731050 PMC8788679

[19] Sheng H, Qi L, Sui Y, Li YZ, Yu LZ, Zhang K, Xu JZ, Wang PM, Zheng DQ. 2019. Mapping chromosomal instability induced by small-molecular therapeutics in a yeast model. Appl Microbiol Biotechnol 103:4869–4880. doi:10.1007/s00253-019-09845-5 31053912

[20] Degtyareva NP, Saini N, Sterling JF, Placentra VC, Klimczak LJ, Gordenin DA, Doetsch PW. 2019. Mutational signatures of redox stress in yeast single-strand DNA and of aging in human mitochondrial DNA share a common feature. PLoS Biol 17:e3000263. doi:10.1371/journal.pbio.3000263 31067233 PMC6527239

[21] Zhang K, Zheng DQ, Sui Y, Qi L, Petes TD. 2019. Genome-wide analysis of genomic alterations induced by oxidative DNA damage in yeast. Nucleic Acids Res 47:3521–3535. doi:10.1093/nar/gkz027 30668788 PMC6468167

[22] Qi L, Zhang K, Wang YT, Wu JK, Sui Y, Liang XZ, Yu LZ, Wu XC, Wang PM, Xu JZ, Zheng DQ. 2019. Global analysis of furfural-induced genomic instability using a yeast model. Appl Environ Microbiol 85:e01237-19. doi:10.1128/AEM.01237-19 31300396 PMC6715844

[23] Qi L, Zhu Y-X, Wang Y-K, Tang X-X, Li K-J, He M, Sui Y, Wang P-M, Zheng D-Q, Zhang K. 2023. Nonlethal furfural exposure causes genomic alterations and adaptability evolution in Saccharomyces cerevisiae. Microbiol Spectr 11:e0121623. doi:10.1128/spectrum.01216-23 37395645 PMC10434202

[24] Luttermann T, Rückert C, Wibberg D, Busche T, Schwarzhans J-P, Friehs K, Kalinowski J. 2021. Establishment of a near-contiguous genome sequence of the citric acid producing yeast Yarrowia lipolytica DSM 3286 with resolution of rDNA clusters and telomeres. NAR Genom Bioinform 3:lqab085. doi:10.1093/nargab/lqab085 34661101 PMC8515841

[25] Vierna J, Wehner S, Höner zu Siederdissen C, Martínez-Lage A, Marz M. 2013. Systematic analysis and evolution of 5S ribosomal DNA in metazoans. Heredity (Edinb) 111:410–421. doi:10.1038/hdy.2013.63 23838690 PMC3806018

[26] Gaillardin C, Mekouar M, Neuvéglise C. 2013. Comparative genomics of *Yarrowia lipolytica*, p 1–30. In Barth G (ed), Yarrowia lipolytica: genetics, genomics, and physiology. Springer, Berlin Heidelberg, Berlin, Heidelberg.

[27] Ullu E, Murphy S, Melli M. 1982. Human 7SL RNA consists of a 140 nucleotide middle-repetitive sequence inserted in an alu sequence. Cell 29:195–202. doi:10.1016/0092-8674(82)90103-9 6179628

[28] He F, Yaver D, Beckerich J-M, Ogrydziak D, Gaillardin C. 1990. The yeast Yarrowia lipolytica has two, functional, signal recognition particle 7S RNA genes. Curr Genet 17:289–292. doi:10.1007/BF00314874 2160331

[29] Yan H, Bu P. 2021. Non-coding RNA in cancer. Essays Biochem 65:625–639. doi:10.1042/EBC20200032 33860799 PMC8564738

[30] Pomraning KR, Bredeweg EL, Kerkhoven EJ, Barry K, Haridas S, Hundley H, LaButti K, Lipzen A, Yan M, Magnuson JK, Simmons BA, Grigoriev IV, Nielsen J, Baker SE. 2018. Regulation of yeast-to-hyphae transition in Yarrowia lipolytica. mSphere 3:e00541-18. doi:10.1128/mSphere.00541-18 30518677 PMC6282006

[31] Sui Y, Qi L, Wu J-K, Wen X-P, Tang X-X, Ma Z-J, Wu X-C, Zhang K, Kokoska RJ, Zheng D-Q, Petes TD. 2020. Genome-wide mapping of spontaneous genetic alterations in diploid yeast cells. Proc Natl Acad Sci U S A 117:28191–28200. doi:10.1073/pnas.2018633117 33106417 PMC7668089

[32] Zhu YO, Siegal ML, Hall DW, Petrov DA. 2014. Precise estimates of mutation rate and spectrum in yeast. Proc Natl Acad Sci U S A 111:E2310–E2318. doi:10.1073/pnas.1323011111 24847077 PMC4050626

[33] Sharp NP, Sandell L, James CG, Otto SP. 2018. The genome-wide rate and spectrum of spontaneous mutations differ between haploid and diploid yeast. Proc Natl Acad Sci U S A 115:E5046–E5055. doi:10.1073/pnas.1801040115 29760081 PMC5984525

[34] Lin G, Wang Y, Guo L, Ding H, Hu Y, Liang S, Zhang Z, Yang G. 2017. Verification of mutagen function of Zeocin in Nannochloropsis oceanica through transcriptome analysis. J Ocean Univ China 16:501–508. doi:10.1007/s11802-017-3231-x

[35] Zhao B, Li Y, Li C, Yang H, Wang W. 2018. Enhancement of Schizochytrium DHA synthesis by plasma mutagenesis aided with malonic acid and zeocin screening. Appl Microbiol Biotechnol 102:2351–2361. doi:10.1007/s00253-018-8756-4 29356868

[36] Liang S, Zhang Z, Liu H, Guo L, Sun S, Yang G. 2019. Identifying the growth associating genes of Nannochloropsis oceanica by bulked mutant analysis (BMA) and RNA sequencing (BMR-seq). J Appl Phycol 31:3677–3690. doi:10.1007/s10811-019-01867-w

[37] Wu JC, Kozarich JW, Stubbe J. 1985. Mechanism of bleomycin: evidence for a rate-determining 4’-hydrogen abstraction from poly(dA-dU) associated with the formation of both free base and base propenal. Biochemistry 24:7562–7568. doi:10.1021/bi00347a009 2418868

[38] Rabow LE, Stubbe J, Kozarich JW. 1990. Identification and quantitation of the lesion accompanying base release in bleomycin-mediated DNA degradation. J Am Chem Soc 112:3196–3203. doi:10.1021/ja00164a049

[39] Jang IS, Yu BJ, Jang JY, Jegal J, Lee JY. 2018. Improving the efficiency of homologous recombination by chemical and biological approaches in Yarrowia lipolytica. PLoS ONE 13:e0194954. doi:10.1371/journal.pone.0194954 29566071 PMC5864075

[40] Nakamura M, Nunoshiba T, Hiratsu K. 2021. Detection and analysis of UV-induced mutations in the chromosomal DNA of Arabidopsis. Biochem Biophys Res Commun 554:89–93. doi:10.1016/j.bbrc.2021.03.087 33784511

[41] Madzak C, Tréton B, Blanchin-Roland S. 2000. Strong hybrid promoters and integrative expression/secretion vectors for quasi-constitutive expression of heterologous proteins in the yeast Yarrowia lipolytica. J Mol Microbiol Biotechnol 2:207–216. 10939246

[42] Bryan C, Le J, Wei X, Yang K. 2023. Saccharomyces cerevisiae apurinic/apyrimidinic endonuclease 1 repairs abasic site-mediated DNA-peptide/protein cross-links. DNA Repair (Amst) 126:103501. doi:10.1016/j.dnarep.2023.103501 37075541

[43] Martin SK, Wood RD. 2019. DNA polymerase ζ in DNA replication and repair. Nucleic Acids Res 47:8348–8361. doi:10.1093/nar/gkz705 31410467 PMC6895278

[44] Nishant KT, Wei W, Mancera E, Argueso JL, Schlattl A, Delhomme N, Ma X, Bustamante CD, Korbel JO, Gu Z, Steinmetz LM, Alani E. 2010. The baker’s yeast diploid genome is remarkably stable in vegetative growth and meiosis. PLoS Genet 6:e1001109. doi:10.1371/journal.pgen.1001109 20838597 PMC2936533

[45] Chatterjee N, Walker GC. 2017. Mechanisms of DNA damage, repair, and mutagenesis. Environ Mol Mutagen 58:235–263. doi:10.1002/em.22087 28485537 PMC5474181

[46] Lynch M, Sung W, Morris K, Coffey N, Landry CR, Dopman EB, Dickinson WJ, Okamoto K, Kulkarni S, Hartl DL, Thomas WK. 2008. A genome-wide view of the spectrum of spontaneous mutations in yeast. Proc Natl Acad Sci U S A 105:9272–9277. doi:10.1073/pnas.0803466105 18583475 PMC2453693

[47] Marchesi F, Turriziani M, Tortorelli G, Avvisati G, Torino F, De Vecchis L. 2007. Triazene compounds: mechanism of action and related DNA repair systems. Pharmacol Res 56:275–287. doi:10.1016/j.phrs.2007.08.003 17897837

[48] Qi L, Sui Y, Tang X-X, McGinty RJ, Liang X-Z, Dominska M, Zhang K, Mirkin SM, Zheng D-Q, Petes TD. 2023. Shuffling the yeast genome using CRISPR/Cas9-generated DSBs that target the transposable Ty1 elements. PLoS Genet 19:e1010590. doi:10.1371/journal.pgen.1010590 36701275 PMC9879454

[49] Narayanan DL, Saladi RN, Fox JL. 2010. Ultraviolet radiation and skin cancer. Int J Dermatol 49:978–986. doi:10.1111/j.1365-4632.2010.04474.x 20883261

[50] Alves RN, Agustí S. 2021. Oxidative stress in tissues of gilthead seabream (Sparus aurata) and European seabass (Dicentrarchus labrax) juveniles exposed to ultraviolet-B radiation. J Photochem Photobiol 8:100070. doi:10.1016/j.jpap.2021.100070

[51] Waters LS, Minesinger BK, Wiltrout ME, D’Souza S, Woodruff RV, Walker GC. 2009. Eukaryotic translesion polymerases and their roles and regulation in DNA damage tolerance. Microbiol Mol Biol Rev 73:134–154. doi:10.1128/MMBR.00034-08 19258535 PMC2650891

[52] Alexandrov LB, Kim J, Haradhvala NJ, Huang MN, Tian Ng AW, Wu Y, Boot A, Covington KR, Gordenin DA, Bergstrom EN, et al.. 2020. The repertoire of mutational signatures in human cancer. Nature New Biol 578:94–101. doi:10.1038/s41586-020-1943-3 PMC7054213 32025018

[53] Brash DE. 2015. UV signature mutations. Photochem Photobiol 91:15–26. doi:10.1111/php.12377 25354245 PMC4294947

[54] Maiorano D, El Etri J, Franchet C, Hoffmann JS. 2021. Translesion synthesis or repair by specialized DNA polymerases limits excessive genomic instability upon replication stress. Int J Mol Sci 22:3924. doi:10.3390/ijms22083924 33920223 PMC8069355

[55] Menck CFM, Galhardo RS, Quinet A. 2024. The accurate bypass of pyrimidine dimers by DNA polymerase eta contributes to ultraviolet-induced mutagenesis. Mutat Res 828:111840. doi:10.1016/j.mrfmmm.2023.111840 37984186

[56] Ikehata H, Ono T. 2011. The mechanisms of UV mutagenesis. J Radiat Res 52:115–125. doi:10.1269/jrr.10175 21436607

[57] Chea J, Zhang S, Zhao H, Zhang Z, Lee EYC, Darzynkiewicz Z, Lee M. 2012. Spatiotemporal recruitment of human DNA polymerase delta to sites of UV damage. Cell Cycle 11:2885–2895. doi:10.4161/cc.21280 22801543 PMC3419061

[58] Hu CW, Chen CM, Ho HH, Chao MR. 2012. Simultaneous quantification of methylated purines in DNA by isotope dilution LC-MS/MS coupled with automated solid-phase extraction. Anal Bioanal Chem 402:1199–1208. doi:10.1007/s00216-011-5559-1 22094591

[59] Wyatt MD, Pittman DL. 2006. Methylating agents and DNA repair responses: methylated bases and sources of strand breaks. Chem Res Toxicol 19:1580–1594. doi:10.1021/tx060164e 17173371 PMC2542901

[60] Loechler EL, Green CL, Essigmann JM. 1984. In vivo mutagenesis by O6-methylguanine built into a unique site in a viral genome. Proc Natl Acad Sci U S A 81:6271–6275. doi:10.1073/pnas.81.20.6271 6093094 PMC391905

[61] McIntyre J. 2020. Polymerase iota - an odd sibling among Y family polymerases. DNA Repair (Amst) 86:102753. doi:10.1016/j.dnarep.2019.102753 31805501

[62] Jiang G, Wang J, Zhao D, Chen X, Pu S, Zhang C, Li J, Li Y, Yang J, Li S, Liao X, Ma H, Ma Y, Zhou Z, Bi C, Zhang X. 2021. Molecular mechanism of the cytosine CRISPR base editing process and the roles of translesion DNA polymerases. ACS Synth Biol 10:3353–3358. doi:10.1021/acssynbio.1c00293 34851089

[63] Bolzán AD, Bianchi MS. 2018. DNA and chromosome damage induced by bleomycin in mammalian cells: an update. Mutat Res Rev Mutat Res 775:51–62. doi:10.1016/j.mrrev.2018.02.003 29555029

[64] Murray V, Chen JK, Tanaka MM. 2016. The genome-wide DNA sequence specificity of the anti-tumour drug bleomycin in human cells. Mol Biol Rep 43:639–651. doi:10.1007/s11033-016-3998-7 27188426

[65] Chen J, Stubbe J. 2005. Bleomycins: towards better therapeutics. Nat Rev Cancer 5:102–112. doi:10.1038/nrc1547 15685195

[66] Boger DL, Cai H. 1999. Bleomycin: synthetic and mechanistic studies. Angew Chem Int Ed Engl 38:448–476. doi:10.1002/(SICI)1521-3773(19990215)38:4<448::AID-ANIE448>3.0.CO;2-W 29711767

[67] Shang J, Qiao Y, Mao G, Qian L, Liu G, Wang H. 2021. Bleomycin-Fe(II) agent with potentiality for treating drug-resistant H1N1 influenza virus: a study using electrochemical RNA beacons. Anal Chim Acta 1180:338862. doi:10.1016/j.aca.2021.338862 34538316

[68] Povirk LF, Bennett RA, Wang P, Swerdlow PS, Austin MJ. 1994. Single base-pair deletions induced by bleomycin at potential double-strand cleavage sites in the aprt gene of stationary phase Chinese hamster ovary D422 cells. J Mol Biol 243:216–226. doi:10.1006/jmbi.1994.1649 7523683

[69] Yu Y, Inamdar KV, Turner K, Jackson-Cook CK, Povirk LF. 2002. Base substitutions, targeted single-base deletions, and chromosomal translocations induced by bleomycin in plateau-phase mammary epithelial cells. Radiat Res 158:327–338. doi:10.1667/0033-7587(2002)158[0327:bstsbd]2.0.co;2 12175310

[70] Selvaraj S, Feist WN, Viel S, Vaidyanathan S, Dudek AM, Gastou M, Rockwood SJ, Ekman FK, Oseghale AR, Xu L, Pavel-Dinu M, Luna SE, Cromer MK, Sayana R, Gomez-Ospina N, Porteus MH. 2024. High-efficiency transgene integration by homology-directed repair in human primary cells using DNA-PKcs inhibition. Nat Biotechnol 42:731–744. doi:10.1038/s41587-023-01888-4 37537500

[71] Lukaszewicz A, Lange J, Keeney S, Jasin M. 2021. De novo deletions and duplications at recombination hotspots in mouse germlines. Cell 184:5970–5984. doi:10.1016/j.cell.2021.10.025 34793701 PMC8616837

[72] Bennett EP, Petersen BL, Johansen IE, Niu Y, Yang Z, Chamberlain CA, Met Ö, Wandall HH, Frödin M. 2020. INDEL detection, the 'Achilles heel' of precise genome editing: a survey of methods for accurate profiling of gene editing induced indels. Nucleic Acids Res 48:11958–11981. doi:10.1093/nar/gkaa975 33170255 PMC7708060

[73] Huang ME, Qin Y, Shang Y, Hao Q, Zhan C, Lian C, Luo S, Liu LD, Zhang S, Zhang Y, et al.. 2024. C-to-G editing generates double-strand breaks causing deletion, transversion and translocation. Nat Cell Biol 26:294–304. doi:10.1038/s41556-023-01342-2 38263276

[74] Nelson JR, Lawrence CW, Hinkle DC. 1996. Deoxycytidyl transferase activity of yeast REV1 protein. Nat New Biol 382:729–731. doi:10.1038/382729a0 8751446

[75] Haracska L, Unk I, Johnson RE, Johansson E, Burgers PM, Prakash S, Prakash L. 2001. Roles of yeast DNA polymerases delta and zeta and of Rev1 in the bypass of abasic sites. Genes Dev 15:945–954. doi:10.1101/gad.882301 11316789 PMC312678

[76] Chen D, Gervai JZ, Póti Á, Németh E, Szeltner Z, Szikriszt B, Gyüre Z, Zámborszky J, Ceccon M, d’Adda di Fagagna F, Szallasi Z, Richardson AL, Szüts D. 2022. BRCA1 deficiency specific base substitution mutagenesis is dependent on translesion synthesis and regulated by 53BP1. Nat Commun 13:226. doi:10.1038/s41467-021-27872-7 35017534 PMC8752635

[77] Gyüre Z, Póti Á, Németh E, Szikriszt B, Lózsa R, Krawczyk M, Richardson AL, Szüts D. 2023. Spontaneous mutagenesis in human cells is controlled by REV1-Polymerase ζ and PRIMPOL. Cell Rep 42:112887. doi:10.1016/j.celrep.2023.112887 37498746

[78] Wang Q, Cui K, Espin-Garcia O, Cheng D, Qiu X, Chen Z, Moore M, Bristow RG, Xu W, Der S, Liu G. 2013. Resistance to bleomycin in cancer cell lines is characterized by prolonged doubling time, reduced DNA damage and evasion of G2/M arrest and apoptosis. PLoS ONE 8:e82363. doi:10.1371/journal.pone.0082363 24349265 PMC3857806

[79] Aouida M, Pagé N, Leduc A, Peter M, Ramotar D. 2004. A genome-wide screen in Saccharomyces cerevisiae reveals altered transport as a mechanism of resistance to the anticancer drug bleomycin. Cancer Res 64:1102–1109. doi:10.1158/0008-5472.can-03-2729 14871844

[80] Primo C, Ferri-Blázquez A, Loewith R, Yenush L. 2017. Reciprocal regulation of target of rapamycin complex 1 and potassium accumulation. J Biol Chem 292:563–574. doi:10.1074/jbc.M116.746982 27895122 PMC5241732

[81] Gietz RD, Schiestl RH. 2007. Large-scale high-efficiency yeast transformation using the LiAc/SS carrier DNA/PEG method. Nat Protoc 2:38–41. doi:10.1038/nprot.2007.15 17401336

[82] Li H, Durbin R. 2009. Fast and accurate short read alignment with Burrows-Wheeler transform. Bioinformatics 25:1754–1760. doi:10.1093/bioinformatics/btp324 19451168 PMC2705234

[83] Li H, Handsaker B, Wysoker A, Fennell T, Ruan J, Homer N, Marth G, Abecasis G, Durbin R, 1000 Genome Project Data Processing Subgroup. 2009. The sequence alignment/map format and SAMtools. Bioinformatics 25:2078–2079. doi:10.1093/bioinformatics/btp352 19505943 PMC2723002

[84] Koboldt DC, Zhang Q, Larson DE, Shen D, McLellan MD, Lin L, Miller CA, Mardis ER, Ding L, Wilson RK. 2012. VarScan 2: somatic mutation and copy number alteration discovery in cancer by exome sequencing. Genome Res 22:568–576. doi:10.1101/gr.129684.111 22300766 PMC3290792

[85] Zheng DQ, Zhang K, Wu XC, Mieczkowski PA, Petes TD. 2016. Global analysis of genomic instability caused by DNA replication stress in Saccharomyces cerevisiae. Proc Natl Acad Sci U S A 113:E8114–E8121. doi:10.1073/pnas.1618129113 27911848 PMC5167146

[86] Bankevich A, Nurk S, Antipov D, Gurevich AA, Dvorkin M, Kulikov AS, Lesin VM, Nikolenko SI, Pham S, Prjibelski AD, Pyshkin AV, Sirotkin AV, Vyahhi N, Tesler G, Alekseyev MA, Pevzner PA. 2012. SPAdes: a new genome assembly algorithm and its applications to single-cell sequencing. J Comput Biol 19:455–477. doi:10.1089/cmb.2012.0021 22506599 PMC3342519

[87] Kent WJ. 2002. BLAT--the BLAST-like alignment tool. Genome Res 12:656–664. doi:10.1101/gr.229202 11932250 PMC187518

[88] Hirota K, Sato T, Hashimoto Y, Yoshioka H, Ohtomo N, Ishihara H, Matsuki A. 1999. Relaxant effect of magnesium and zinc on histamine-induced bronchoconstriction in dogs. Crit Care Med 27:1159–1163. doi:10.1097/00003246-199906000-00042 10397222

[89] Schneider TD, Stephens RM. 1990. Sequence logos: a new way to display consensus sequences. Nucleic Acids Res 18:6097–6100. doi:10.1093/nar/18.20.6097 2172928 PMC332411

[90] Schwartz CM, Hussain MS, Blenner M, Wheeldon I. 2016. Synthetic RNA polymerase III promoters facilitate high-efficiency CRISPR-Cas9-mediated genome editing in Yarrowia lipolytica. ACS Synth Biol 5:356–359. doi:10.1021/acssynbio.5b00162 26714206

