# Supplementary material for: Patterns of spontaneous and induced genomic alterations in Yarrowia lipolytica
Source: Appl Environ Microbiol. 2024 Dec 23;91(1):e01678-24. doi: 10.1128/aem.01678-24 (PMC11784153; doi:10.1128/aem.01678-24)
Supplement: Supplemental material — Tables S1 to S3; Fig. S1 to S3. [file aem.01678-24-s0002.docx]

**The patterns of spontaneous and induced genomic alterations in *Yarrowia lipolytica***

Yuan-Ru Xiong^1,2^, Yuan-Chun Fang^2^, Min He^2^, Ke-Jing Li^2^, Lei Qi^2^, Yang Sui^2^, Ke Zhang^3^, Xue-Chang Wu^3^, Liang Meng^4^, Ou Li^1,^*, Dao-Qiong Zheng^2,^*

^1^ College of Life Sciences and Medicine, Zhejiang Sci-Tech University, Hangzhou, 310018, China

^2^ Ocean College, Zhejiang University, Zhoushan 321006, China

^3^ College of Life Science, Zhejiang University, Hangzhou 310030, China

^4^ BGI Research, Sanya 572025, China

*Correspondence: Dao-Qiong Zheng or Ou Li

Address: Room 377, Marine Science Building, No.1 Zheda Road, Dinghai District, Zhoushan, Zhejiang, China, 316021

E-mail: [zhengdaoqiong@zju.edu.cn](mailto:zhengdaoqiong@zju.edu.cn) (DZ) or ouli@zstu.edu.cn (OL)

Phone No.: +86 580 2092277

**Supplemental text**

Strain PPF was generated by integrating *CAS9*-*LEU2* into the genome of strain PO1f. The *CAS9*-*LEU2* cassette was amplified from pCRISPRyl using primers listed in Table S1. To facilitate integration, Zeta_NotI-up and Zeta_NotI-down regions were amplified from the plasmid GGE067 (https://www.addgene.org/120784/) (1). These fragments were combined with the *CAS9-LEU2* cassette through overlap PCR to construct the expression cassette Zeta_NotⅠ-up-Cas9-LEU2-Zeta_NotI-down. Transformation of this cassette into PO1f allowed for the integration of *CAS9* into the genome, resulting in the strain PPF.

To delete the *LYS9* gene in strain PPF, the plasmid pU2gRLYS9 was constructed using the plasmid pU2gR as a backbone (Fig. S1). The upstream (using primers L9U-s and L9U-a) and downstream (using primers L9D-s and L9D-a) homology arms of *LYS9* were amplified from the PPF genome. These two fragments were then ligated through overlap PCR and transformed into PPF along with the pU2gRLYS9 plasmid. Correct transformants were selected and purified through multiple passages on uracil-containing media to eliminate the pU2gRLYS9 plasmid, resulting in the strain PPY3.

Strain PPW was generated by randomly inserting *LYS9* and *URA3* into the genome of strain PPY3, as described below. The full open reading frame of *LYS9* was amplified using primers Lys9-s and Lys9-a, and *URA3* was amplified using primers O-ura-S and URA3YL-A, both using the W29 genome as the template. These two fragments were co-transformed into PPY3. Transformants were selected by growing them on a nutritional deficiency screening medium lacking lysine and uracil.

**Table S1.** Primers used in this study.

| **Primer** | **Sequence (5'→3')** | **Purpose** |
| --- | --- | --- |
| Zetaup-s | gcggccgctgtcgggaaccg | Amplification of *Zeta_NotI-up* for the expression cassette *Zeta_NotI-up-Cas9-LEU2-Zeta_NotI-down* |
| Zetaup-a | tctagcaaagtgctttgtgc |  |
| Zetadn-s | catgtgtaacactcgctctg | Amplification of *Zeta_NotI-down* for the expression cassette *Zeta_NotI-up-Cas9-LEU2-Zeta_NotI-down* |
| Zetdn-a | gcggccgcactgaagggctt |  |
| ZUC9-s | gcacaaagcactttgctagaatggataagaaatactccattggcc | Amplification of *Cas9* for the expression cassette *Zeta_NotI-up-Cas9-LEU2-Zeta_NotI-down* |
| cas9T-A | gcaaattaaagccttcgagcgtc |  |
| cas9+pleu-S | acgctcgaaggctttaatttgcagatctgttcggaaatcaacgg | Amplification of *LEU2* for the expression cassette *Zeta_NotI-up-Cas9-LEU2-Zeta_NotI-down* |
| tleu+ZD-A | ccagagcgagtgttacacatgtcgacaccatatcatataaaactaacaat |  |
| vCas9-s | GCAGAAGAAGGCTATTGTGGAT | Verification of correct insertion of the expression cassette *Zeta_NotI-up-Cas9-LEU2-Zeta_NotI-down* |
| vCas9-a | GTAGTCGGAGAGTCGGTTGAT |  |
| LYS9up-s | TGTTGGCGGATGGCTATA | Amplification of the upstream homology arm of *LYS9* |
| LYS9up-a | CGCAAGCTGGCTAATGTACTCTGGCATAAGGTT |  |
| LYS9dn-s | GCCAGAGTACATTAGCCAGCTTGCGGTGAA | Amplification of the downstream homology arm of *LYS9* |
| LYS9dn-a | GCTCATGTACTCCAGATCCTTC |  |
| vdlys9-s | AATGGAGTTGCTTCTGTGTTCA | Verification of the sequence loss of *LYS9* |
| vdlys9-a | TAGTTAGGTGGTGATGATGATTCG |  |
| O-ura-S | tcgcttcggataactcctgcta | Amplification of *URA3* gene for the construction of PPW |
| URA3YL-A | tctgaattccgagaaacacaac |  |
| Lys9-s | CTCGGTCCACGTTCGTCA | Amplification of *LYS9* as a reporter gene for the deletion cassette and for construction of PPW |
| Lys9-a | CCACAGTCGGCTTTAGAGGA |  |
| del1up-s | gattgaagaccctgcctttgat | Amplification of the upstream homology arm of *YALI1_E14028g* for the deletion cassette |
| del1up-a | GCATGACGAACGTGGACCGAGcgccgaccaccacaatatc |  |
| del1dn-s | AAAATTCCTCTAAAGCCGACTGTGGagttatctcagtctcggtctca | Amplification of the downstream homology arm of *YALI1_E14028g* for the deletion cassette |
| del1dn-a | ggctactcgttatgttccacta |  |
| del4up-s | catctatggtctaagcggtcat | Amplification of the upstream homology arm of *YALI1_F15859g* for the deletion cassette |
| del4up-a | GCATGACGAACGTGGACCGAGgcaatggttggtaactgtgaa |  |
| del4dn-s | AAAATTCCTCTAAAGCCGACTGTGGcacctgtgtcaatcaagaagag | Amplification of the downstream homology arm of *YALI1_F15859g* for the deletion cassette |
| del4dn-a | gttcaacatcacgagcagtt |  |
| del6up-s | agtcgcgtgtgatttctttgat | Amplification of the upstream homology arm of *YALI1_A00538g* |
| del6up-a | GCATGACGAACGTGGACCGAGgacgagggctctggttcttat |  |
| del6dn-s | AAAATTCCTCTAAAGCCGACTGTGGaattcaagtggaagacgctga | Amplification of the downstream homology arm of *YALI1_A00538g* |
| del6dn-a | acctccgacctaccttctatg |  |
| del7up-s | ccgagaggaatggcgagtt | Amplification of the upstream homology arm of *YALI1_E21053g* |
| del7up-a | GCATGACGAACGTGGACCGAGgcttgggaatggcgagatg |  |
| del7dn-s | AAAATTCCTCTAAAGCCGACTGTGGcgattctagcatacggcaactc | Amplification of the downstream homology arm of *YALI1_E21053g* |
| del7dn-a | acacgacgactcgctcaac |  |
| del8up-s | gccttcctgtacttatgtcact | Amplification of the upstream homology arm of *YALI1_B18292g* |
| del8up-a | GCATGACGAACGTGGACCGAGagcatccacaatcatctccatt |  |
| del8dn-s | AAAATTCCTCTAAAGCCGACTGTGGtgtgtcgtagaagtgctggta | Amplification of the downstream homology arm of *YALI1_B18292g* |
| del8dn-a | acagaggaaggcgaacagta |  |
| del9up-s | gaggcgatcaccaggacat | Amplification of the upstream homology arm of *YALI1_B25553g* |
| del9up-a | GCATGACGAACGTGGACCGAGttcttggctgacacgacaac |  |
| del9dn-s | AAAATTCCTCTAAAGCCGACTGTGGgctagactctaagaccgctgaa | Amplification of the downstream homology arm of *YALI1_B25553g* |
| del9dn-a | catagaccgtgccagatgtagt |  |
| del10up-s | cctcgtgctatctctgaccat | Amplification of the upstream homology arm of *YALI1_F26427g* |
| del10up-a | GCATGACGAACGTGGACCGAGcgattccgaacagccaactt |  |
| del10dn-s | AAAATTCCTCTAAAGCCGACTGTGGttgccgcttgtctcttgtc | Amplification of the downstream homology arm of *YALI1_F26427g* |
| del10dn-a | acgccagaactaatatccacat |  |
| del12up-s | atcacccaccctcagcaatt | Amplification of the upstream homology arm of *YALI1_C11234g* |
| del12up-a | GCATGACGAACGTGGACCGAGcgaacggcatcaactctatcc |  |
| del12dn-s | AAAATTCCTCTAAAGCCGACTGTGGagaagcccgagagaagatgag | Amplification of the downstream homology arm of *YALI1_C11234g* |
| del12dn-a | ttctgctatggttccgttatgg |  |
| vd1-s | cgagatgcgaggtcaagaac | Verification of *YALI1_E14028g* deletion |
| vd1-a | ttccgtcgtctgccgtatt |  |
| vd4-s | cttcatcttcgccatcctcatc | Verification of correct insertion of *YALI1_F15859g* deletion cassette |
| vd4-a | gctcttagtcggtcaccattg |  |
| vd6-s | ttagccgccttccgtatctg | Verification of *YALI1_A00538g* deletion |
| vd6-a | cctccgacctaccttctatgc |  |
| vd7-s | cggttcaacttcggctcatc | Verification of *YALI1_E21053g* deletion |
| vd7-a | ctcgcaaggtcactctccat |  |
| vd8-s | atgtggcgtaggttagagtgt | Verification of correct insertion of *YALI1_B18292g* deletion cassette |
| vd8-a | tcgttgctattgcttgatgtga |  |
| vd9-s | cctccttgtctgttgcttgtc | Verification of *YALI1_B25553g* deletion |
| vd9-a | gtcgcttccacaccttcca |  |
| vd10-s | cctcgtgctatctctgaccat | Verification of *YALI1_F26427g* deletion |
| vd10-a | cgctctcggacattcttagtg |  |
| vd12-s | cggagtgtgaacaagagtaacc | Verification of *YALI1_C11234g* deletion |
| vd12-a | cgctgccaaccaaccaatc |  |
| delku70up-s | ctcactcttaggctcgcttaga | Amplification of the upstream homology arm of *KU70* |
| delku70up-a | TGCATGACGAACGTGGACCGAGggcaccgaacagcataacg |  |
| delku70dn-s | CCAAAAATTCCTCTAAAGCCGACTGTGGtctcggcttccacaattcct | Amplification of the downstream homology arm of *KU70* |
| delku70dn-a | acatcgcacagacaccagta |  |
| vdku70-s | ACCGACAGAGATACAGCAGAT | Verification of *KU70* deletion |
| vdku70-a | CGTCCAACAACAGCGATGAG |  |
| delrev1up-s | CGCAACGACGGATTCTACt | Amplification of the upstream homology arm of *REV1* |
| delrev1up-a | GCATGACGAACGTGGACCGAGAGGCATATTGGCATAGAGTTCA |  |
| delrev1dn-s | CCAAAAATTCCTCTAAAGCCGACTGTGGGATtggagactggagaagaagg | Amplification of the downstream homology arm of *REV1* |
| delrev1dn-a | AAGTGAACTGACGGCAACTC |  |
| vdrev1-s | agtgcgagtgctagtatgagt | Verification of *REV1* deletion |
| vdrev1-a | tgcttgcgttgcgaatctt |  |
| delrev3up-s | CGTCGTTAGCAATGTCATCCT | Amplification of the upstream homology arm of *REV3* |
| delrev3up-a | GCATGACGAACGTGGACCGAGGCAAGTCTCCCGTTCGTTT |  |
| delrev3dn-s | AAATTCCTCTAAAGCCGACTGTGGGGAGCCGTTCATAGCAGAC | Amplification of the downstream homology arm of *REV3* |
| delrev3dn-a | GTAGCAGTAGCAACAGCAGTA |  |
| vdrev3-s | gtatcttgcgaatgcgactaca | Verification of *REV3* deletion |
| vdrev3-a | gcggaaggctctattgactct |  |
| delrad30up-s | TCTGTTAAGCCGCTAATATCGT | Amplification of the upstream homology arm of *RAD30* |
| delrad30up-a | AGTGCATGACGAACGTGGACCGAGGGACCCTGGAAACCCTCAA |  |
| delrad30dn-s | CCAAAAATTCCTCTAAAGCCGACTGTGGCTTCCGTCTTGAACAGCAGTA | Amplification of the downstream homology arm of *RAD30* |
| delrad30dn-a | TCAAGTGTCAGGCTCAGTCT |  |
| vdrad30-s | aaggctcattggagaagattgg | Verification of *RAD30* deletion |
| vdrad30-a | atactgctgttcaagacggaag |  |
| amp-Bone-S | acgtcaggtggcacttttcgg | Amplification of sequence AmpR-ori for the construction of pU2gR |
| ampCR-A | ggcgtaatcatggtcatagctg |  |
| amp-cen-S | cagctatgaccatgattacgccAAGCTTccctttcatcaaatttagggatgcca | Amplification of sequence CEN for the construction of pU2gR |
| Cas9 bone-A | ggccctttcgtctcgcgcgtt |  |
| SCRp-S1-g1-2 | ccgaaacgcgcgagacgaaagggccccccagttgcaaaagttgacacaa | Amplification of sequence Promoter SCR1-tRNA-Gly-gRNA scaffold for the construction of pU2gR |
| scaffold-A1-g1 | gtatagcaggagttatccgaagcgaGTCGACgcggccgcttcgactctagag |  |
| URAt-S2-g2 | gttgtgtttctcggaattcagaCCATGgcatactactgtatattcaagcaagt | Amplification of sequence ARS-gRNA scaffold-tRNA-Gly-Promoter SCR1 for the construction of pU2gR |
| scrP-A2-g2 | ttccccgaaaagtgccacctgacgtGGATCCccccagttgcaaaagttgacacaa |  |
| O-ura-S | tcgcttcggataactcctgcta | Amplification of sequence URA3 for the construction of pU2gR |
| URA3YL-A | tctgaattccgagaaacacaac |  |
| KU70-g1-S | gtgccatgttgataaaatgggttttagagctagaaatagcaagtt | Amplification of sequence sgRNA1 KU70-gRNA scaffold-URA3-ARS-gRNA scaffold-sgRNA2 KU70 for the construction of pU2gRKU70 |
| KU70-g2-A | caggccgtcactattgaaaggttttagagctagaaatagcaagtt |  |
| KU70-g2-S | ctttcaatagtgacggcctgcaacctgcgccgacccgg | Amplification of sequence sgRNA2 KU70-tRNA-Gly-Promoter SCR1-AmpR-ori-CEN-Promoter SCR1-tRNA-Gly-sgRNA1 KU70 for the construction of pU2gRKU70 |
| KU70-g1-A | ccattttatcaacatggcaccaacctgcgccgacccgg |  |
| RAD30-g1-S | aagtgcacaatgcaggccatgttttagagctagaaatagcaagtt | Amplification of sequence sgRNA1 RAD30-gRNA scaffold-URA3-ARS-gRNA scaffold-sgRNA2 RAD30 for the construction of pU2gRRAD30 |
| RAD30-g2-A | caaaaagtaccccatcgatggttttagagctagaaatagcaagtt |  |
| RAD30-g2-S | catcgatggggtactttttgcaacctgcgccgacccgg | Amplification of sequence sgRNA2 RAD30-tRNA-Gly-Promoter SCR1-AmpR-ori-CEN-Promoter SCR1-tRNA-Gly-sgRNA1 RAD30 for the construction of pU2gRRAD30 |
| RAD30-g1-A | atggcctgcattgtgcacttcaacctgcgccgacccgg |  |
| REV1-g1-S | agatacatacctcagcccgcgttttagagctagaaatagcaagtt | Amplification of sequence sgRNA1 REV1-gRNA scaffold-URA3-ARS-gRNA scaffold-sgRNA2 REV1 for the construction of pU2gRREV1 |
| REV1-g2-A | caaaagtccagattggtcgggttttagagctagaaatagcaagtt |  |
| REV1-g2-S | ccgaccaatctggacttttgcaacctgcgccgacccgg | Amplification of sequence sgRNA2 REV1-tRNA-Gly-Promoter SCR1-AmpR-ori-CEN-Promoter SCR1-tRNA-Gly-sgRNA1 REV1 for the construction of pU2gRREV1 |
| REV1-g1-A | gcgggctgaggtatgtatctcaacctgcgccgacccgg |  |
| REV3-g1-S | tatttactactcacgggtcagttttagagctagaaatagcaagtt | Amplification of sequence sgRNA1 REV3-gRNA scaffold-URA3-ARS-gRNA scaffold-sgRNA2 REV3 for the construction of pU2gRREV3 |
| REV3-g2-A | atatccgcagcactggaaccgttttagagctagaaatagcaagtt |  |
| REV3-g2-S | ggttccagtgctgcggatatcaacctgcgccgacccgg | Amplification of sequence sgRNA2 REV3-tRNA-Gly-Promoter SCR1-AmpR-ori-CEN-Promoter SCR1-tRNA-Gly-sgRNA1 REV3 for the construction of pU2gRREV3 |
| REV3-g1-A | tgacccgtgagtagtaaatacaacctgcgccgacccgg |  |
| 1-g1-S | tagtgtagacatgagtggcggttttagagctagaaatagcaagtt | Amplification of sequence sgRNA1 YALI1_E14028g-gRNA scaffold-URA3-ARS-gRNA scaffold-sgRNA2 YALI1_E14028g for the construction of pU2gRE14028 |
| 1-g2-A | agtaataggttccaggagtagttttagagctagaaatagcaagtt |  |
| 1-g2-S | tactcctggaacctattactcaacctgcgccgacccgg | Amplification of sequence sgRNA2 YALI1_E14028g-tRNA-Gly-Promoter SCR1-AmpR-ori-CEN-Promoter SCR1-tRNA-Gly-sgRNA1 YALI1_E14028g for the construction of pU2gRE14028 |
| 1-g1-A | cgccactcatgtctacactacaacctgcgccgacccgg |  |
| 4-g1-S | agttgttgagccgattacacgttttagagctagaaatagcaagtt | Amplification of sequence sgRNA1 YALI1_F15859g-gRNA scaffold-URA3-ARS-gRNA scaffold-sgRNA2 YALI1_F15859g for the construction of pU2gRF15859 |
| 4-g2-A | caatcatgcagtaaagataggttttagagctagaaatagcaagtt |  |
| 4-g2-S | ctatctttactgcatgattgcaacctgcgccgacccgg | Amplification of sequence sgRNA2 YALI1_F15859g-tRNA-Gly-Promoter SCR1-AmpR-ori-CEN-Promoter SCR1-tRNA-Gly-sgRNA1 YALI1_F15859g for the construction of pU2gRF15859 |
| 4-g1-A | gtgtaatcggctcaacaactcaacctgcgccgacccgg |  |
| 6-g1-S | actcaagtgtgctcctgtctgttttagagctagaaatagcaagtt | Amplification of sequence sgRNA1 YALI1_A00538g-gRNA scaffold-URA3-ARS-gRNA scaffold-sgRNA2 YALI1_A00538g for the construction of pU2gRA00538 |
| 6-g2-A | ctcgtaccaacgcgaccccggttttagagctagaaatagcaagtt |  |
| 6-g2-S | cggggtcgcgttggtacgagcaacctgcgccgacccgg | Amplification of sequence sgRNA2 YALI1_A00538g-tRNA-Gly-Promoter SCR1-AmpR-ori-CEN-Promoter SCR1-tRNA-Gly-sgRNA1 YALI1_A00538g for the construction of pU2gRA00538 |
| 6-g1-A | agacaggagcacacttgagtcaacctgcgccgacccgg |  |
| 7-g1-S | gggtcctgtgagtcattcgagttttagagctagaaatagcaagtt | Amplification of sequence sgRNA1 YALI1_E21053g-gRNA scaffold-URA3-ARS-gRNA scaffold-sgRNA2 YALI1_E21053g for the construction of pU2gRE21053 |
| 7-g2-A | gatacgatttggccatcaaggttttagagctagaaatagcaagtt |  |
| 7-g2-S | cttgatggccaaatcgtatccaacctgcgccgacccgg | Amplification of sequence sgRNA2 YALI1_E21053g-tRNA-Gly-Promoter SCR1-AmpR-ori-CEN-Promoter SCR1-tRNA-Gly-sgRNA1 YALI1_E21053g for the construction of pU2gRE21053 |
| 7-g1-A | tcgaatgactcacaggaccccaacctgcgccgacccgg |  |
| 8-g1-S | ccgcagtaagagtttccacggttttagagctagaaatagcaagtt | Amplification of sequence sgRNA1 YALI1_B18292g-gRNA scaffold-URA3-ARS-gRNA scaffold-sgRNA2 YALI1_B18292g for the construction of pU2gRB18292 |
| 8-g2-A | gagagcccaccgtcgccaaggttttagagctagaaatagcaagtt |  |
| 8-g2-S | cttggcgacggtgggctctccaacctgcgccgacccgg | Amplification of sequence sgRNA2 YALI1_B18292g-tRNA-Gly-Promoter SCR1-AmpR-ori-CEN-Promoter SCR1-tRNA-Gly-sgRNA1 YALI1_B18292g for the construction of pU2gRB18292 |
| 8-g1-A | cgtggaaactcttactgcggcaacctgcgccgacccgg |  |
| 9-g1-S | caagccctcagtgtcaatgtgttttagagctagaaatagcaagtt | Amplification of sequence sgRNA1 YALI1_B25553g-gRNA scaffold-URA3-ARS-gRNA scaffold-sgRNA2 YALI1_B25553g for the construction of pU2gRB25553 |
| 9-g2-A | ctcgcctccaataggatacagttttagagctagaaatagcaagtt |  |
| 9-g2-S | tgtatcctattggaggcgagcaacctgcgccgacccgg | Amplification of sequence sgRNA2 YALI1_B25553g-tRNA-Gly-Promoter SCR1-AmpR-ori-CEN-Promoter SCR1-tRNA-Gly-sgRNA1 YALI1_B25553g for the construction of pU2gRB25553 |
| 9-g1-A | acattgacactgagggcttgcaacctgcgccgacccgg |  |
| 10-g1-S | cgaccctggtacgtgcgatggttttagagctagaaatagcaagtt | Amplification of sequence sgRNA1 YALI1_F26427g-gRNA scaffold-URA3-ARS-gRNA scaffold-sgRNA2 YALI1_F26427g for the construction of pU2gRF26427 |
| 10-g2-A | acgctctcgacatatataccgttttagagctagaaatagcaagtt |  |
| 10-g2-S | ggtatatatgtcgagagcgtcaacctgcgccgacccgg | Amplification of sequence sgRNA2 YALI1_F26427g-tRNA-Gly-Promoter SCR1-AmpR-ori-CEN-Promoter SCR1-tRNA-Gly-sgRNA1 YALI1_F26427g for the construction of pU2gRF26427 |
| 10-g1-A | catcgcacgtaccagggtcgcaacctgcgccgacccgg |  |
| 12-g1-S | gtttccagcgaaacctatgagttttagagctagaaatagcaagtt | Amplification of sequence sgRNA1 YALI1_C11234g-gRNA scaffold-URA3-ARS-gRNA scaffold-sgRNA2 YALI1_C11234g for the construction of pU2gRC11234 |
| 12-g2-A | ctcgaagtacgaaaccatcagttttagagctagaaatagcaagtt |  |
| 12-g2-S | tgatggtttcgtacttcgagcaacctgcgccgacccgg | Amplification of sequence sgRNA2 YALI1_C11234g-tRNA-Gly-Promoter SCR1-AmpR-ori-CEN-Promoter SCR1-tRNA-Gly-sgRNA1 YALI1_C11234g for the construction of pU2gRC11234 |
| 12-g1-A | tcataggtttcgctggaaaccaacctgcgccgacccgg |  |

**Table S2.** The context signatures of base substitutions

| Conditions | Substitution | Site | Ratio/site/base | | | |
| --- | --- | --- | --- | --- | --- | --- |
|  |  |  | A | C | G | T |
| Spontaneous | C>T | -2 | 18% | 23% | 41% | 18% |
|  |  | -1 | 27% | 32% | 5% | 36% |
|  |  | 1 | 18% | 18% | 41% | 23% |
|  |  | 2 | 27% | 18% | 14% | 41% |
| Ultraviolet | C>T | -2 | 23% | 28% | 13% | 36% |
|  |  | -1 | 7% | 34% | 4% | 55% |
|  |  | 1 | 25% | 32% | 15% | 28% |
|  |  | 2 | 35% | 23% | 16% | 26% |
| MMS | A>T | -2 | 17% | 36% | 28% | 19% |
|  |  | -1 | 20% | 34% | 33% | 13% |
|  |  | 1 | 19% | 42% | 31% | 8% |
|  |  | 2 | 45% | 17% | 22% | 16% |
|  | A>C | -2 | 43% | 13% | 26% | 17% |
|  |  | -1 | 17% | 78% | 4% | 0% |
|  |  | 1 | 22% | 30% | 35% | 13% |
|  |  | 2 | 39% | 13% | 35% | 13% |
| Zeocin | T>A | -2 | 9% | 19% | 18% | 54% |
|  |  | -1 | 17% | 6% | 71% | 6% |
|  |  | 1 | 23% | 51% | 5% | 21% |
|  |  | 2 | 19% | 33% | 26% | 22% |
|  | T>G | -2 | 12% | 22% | 12% | 54% |
|  |  | -1 | 7% | 5% | 76% | 12% |
|  |  | 1 | 37% | 39% | 12% | 12% |
|  |  | 2 | 19% | 49% | 27% | 5% |
|  | C>A | -2 | 23% | 26% | 9% | 42% |
|  |  | -1 | 2% | 2% | 87% | 9% |
|  |  | 1 | 8% | 77% | 0% | 15% |
|  |  | 2 | 32% | 19% | 28% | 21% |

**Table S3.** Mononucleotide repeat numbers (length) in the genomes of *Y. lipolytica* and *S. cerevisiae*

| **Yeasts** | **A repeat** | **T repeat** | **C repeat** | **G repeat** | **Total** |
| --- | --- | --- | --- | --- | --- |
| *Y. lipolytica* | 244166  (833881) | 243950  (833181) | 191405  (638839) | 190138  (633811) | 869659  (2939712) |
| *S. cerevisiae* | 228094 (776058) | 227455 (774076) | 63455 (204311) | 62312 (200506) | 581316 (1954951) |


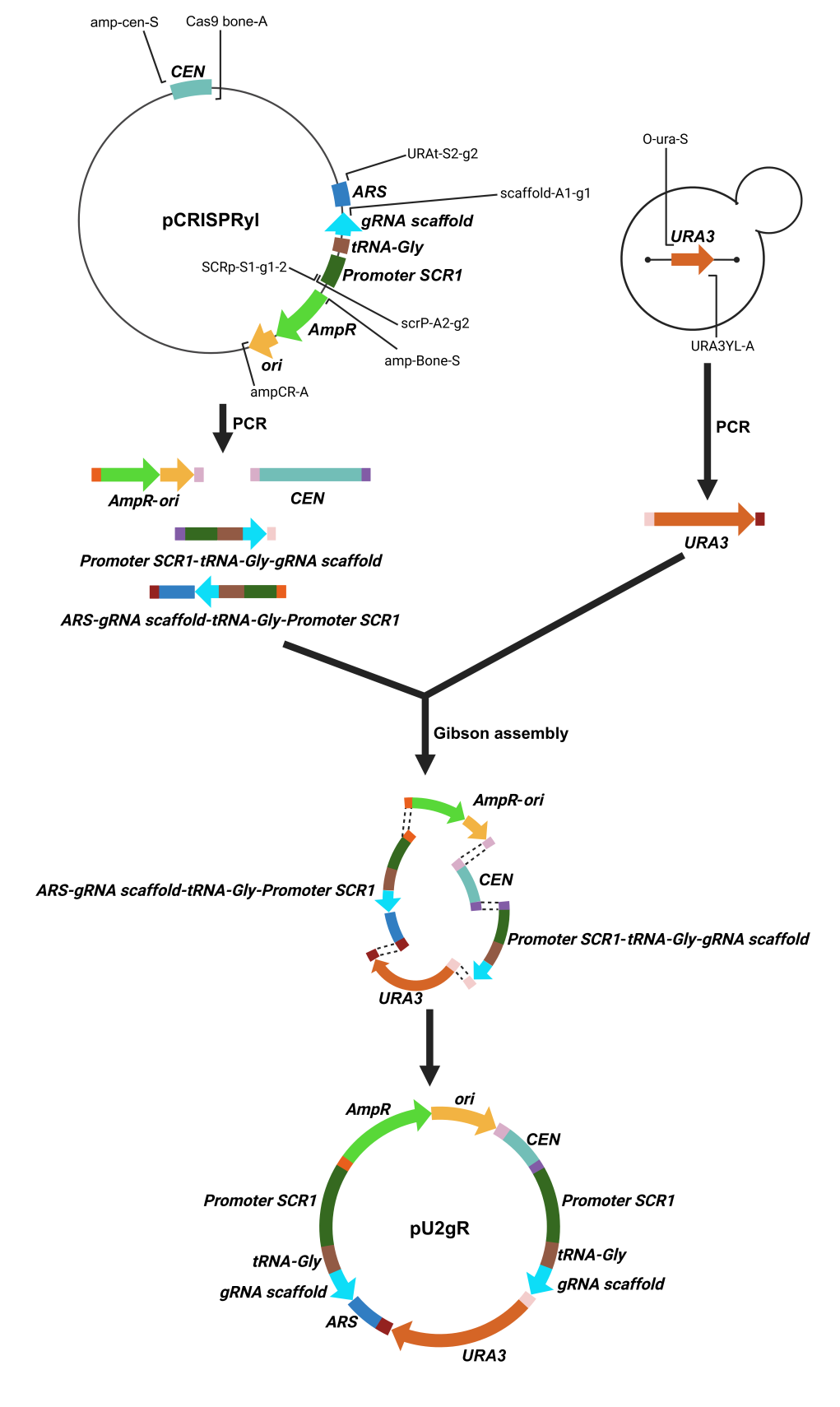


**Fig. S1.** **Construction of plasmid pU2gR**. Using plasmid **pCRISPRyl** as a PCR template, we obtained the following elements: the selection marker ***AmpR***, **SCR1-tRNAGly** promoter, gRNA scaffold, and ***CEN*** and ***ARS*** sequences. A ***URA3*** gene was amplified from the genomic DNA of W29 by PCR. These elements were assembled to generate the plasmid **pU2gR**.


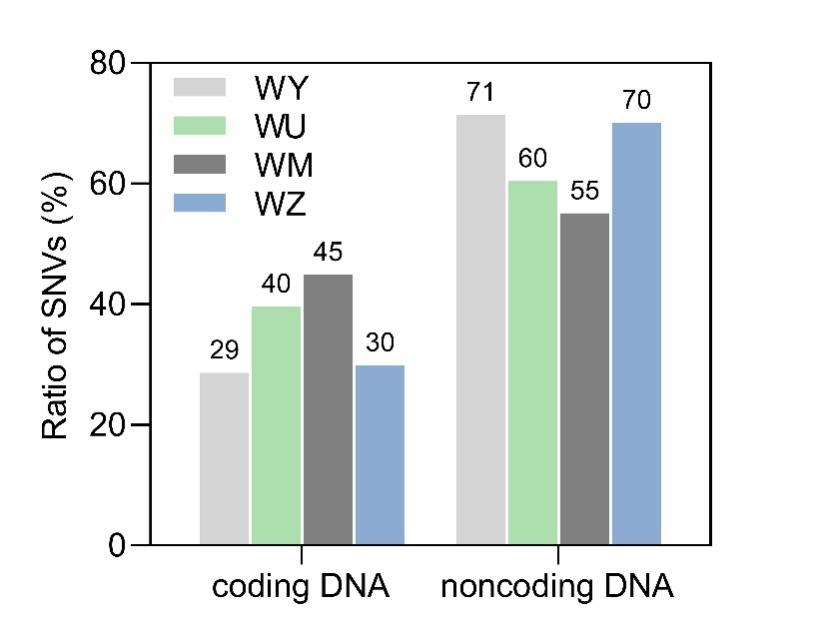


**Fig. S2.** **The ratios of SNVs located on coding and noncoding regions in *Y. lipolytica* genome.** WY, WZ, WU, and WM indicate the isolates subcultured under spontaneous, Zeocin-treated, UV-treated, and MMS-treated conditions.


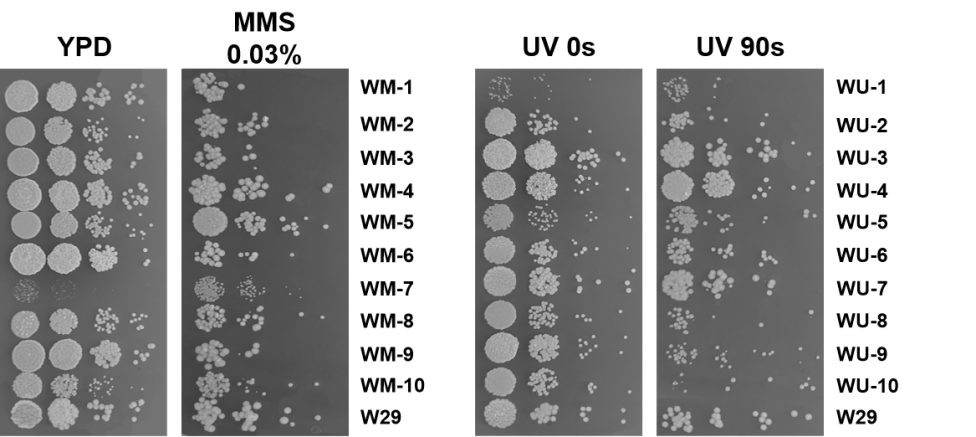


**Fig. S3.** Comparison of stress tolerance of subcultured isolates and their parent strain W29. WM1-10 were isolates subcultured on MMS containing plates, while WU1-10 were isolates treated with UV.

**Reference**

1. Larroude M, Park YK, Soudier P, Kubiak M, Nicaud JM, Rossignol T. 2019. A modular Golden Gate toolkit for *Yarrowia lipolytica* synthetic biology. Microb Biotechnol 12:1249-1259.
